# Suppression of viral RNA polymerase activity is necessary for persistent infection during the transformation of measles virus into SSPE virus

**DOI:** 10.1371/journal.ppat.1011528

**Published:** 2023-07-26

**Authors:** Kento Sakamoto, Miho Konami, Shinra Kameda, Yuto Satoh, Hiroshi Wakimoto, Yoshinori Kitagawa, Bin Gotoh, Da-Peng Jiang, Hak Hotta, Masae Itoh

**Affiliations:** 1 Department of Microbiology, Faculty of Bio-Science, Nagahama Institute of Bio-Science and Technology, Nagahama, Shiga, Japan; 2 Division of Microbiology and Infectious Diseases, Department of Pathology, Shiga University of Medical Science, Otsu, Shiga, Japan; 3 Graduate School of Medicine, Kobe University, Kobe, Hyogo, Japan; University of Texas Medical Branch / Galveston National Laboratory, UNITED STATES

## Abstract

Subacute sclerosing panencephalitis (SSPE) is a fatal neurodegenerative disease caused by measles virus (MV), which typically develops 7 to 10 years after acute measles. During the incubation period, MV establishes a persistent infection in the brain and accumulates mutations that generate neuropathogenic SSPE virus. The neuropathogenicity is closely associated with enhanced propagation mediated by cell-to-cell fusion in the brain, which is principally regulated by hyperfusogenic mutations of the viral F protein. The molecular mechanisms underlying establishment and maintenance of persistent infection are unclear because it is impractical to isolate viruses before the appearance of clinical signs. In this study, we found that the L and P proteins, components of viral RNA-dependent RNA polymerase (RdRp), of an SSPE virus Kobe-1 strain did not promote but rather attenuated viral neuropathogenicity. Viral RdRp activity corresponded to F protein expression; the suppression of RdRp activity in the Kobe-1 strain because of mutations in the L and P proteins led to restriction of the F protein level, thereby reducing cell-to-cell fusion mediated propagation in neuronal cells and decreasing neuropathogenicity. Therefore, the L and P proteins of Kobe-1 did not contribute to progression of SSPE. Three mutations in the L protein strongly suppressed RdRp activity. Recombinant MV harboring the three mutations limited viral spread in neuronal cells while preventing the release of infectious progeny particles; these changes could support persistent infection by enabling host immune escape and preventing host cell lysis. Therefore, the suppression of RdRp activity is necessary for the persistent infection of the parental MV on the way to transform into Kobe-1 SSPE virus. Because mutations in the genome of an SSPE virus reflect the process of SSPE development, mutation analysis will provide insight into the mechanisms underlying persistent infection.

## Author summary

Subacute sclerosing panencephalitis (SSPE) is a rare but fatal disease in apparently healthy children, which develops 7 to 10 years after acute measles. Measles virus (MV) persistently infects the brain, accumulates mutations, and transforms into neuropathogenic SSPE virus. The World Health Organization is promoting vaccination against MV to achieve global eradication of measles; this is also the most effective approach for prevention of SSPE. The live MV vaccine strains do not cause SSPE and all SSPE viruses isolated thus far have been derived from wild-type MV strains, but it remains unclear why live MV vaccine strains do not cause SSPE. Recently hyperfusogenic mutations of the MV F protein were proved to account for the enhanced propagation of the virus in the brain leading to the neurovirulence. In this study we showed that suppression of viral RNA polymerase is implicated in the establishment of persistent wild-type MV infection within neurons. Further investigations of the mechanisms by which wild-type MV transforms into SSPE virus will shed light on the reason why MV vaccine strains do not acquire neurovirulence.

## Introduction

Measles is a highly contagious acute infectious disease caused by measles virus (MV). Despite the availability of a safe and effective attenuated live virus vaccine, a worldwide resurgence of measles occurred between 2017 and 2019; the global incidence of measles reached 120 per million in 2019, and there were approximately 207,500 deaths [1]. Although low case numbers were reported in 2020 and 2021 because of the COVID-19 pandemic, delays and reductions in both vaccination and surveillance programs have increased the risk of additional measles resurgence in the near future [2,3]. MV also causes chronic persistent central nervous system (CNS) infection, subacute sclerosing panencephalitis (SSPE) in fully immunocompetent persons, which occurs 7 to 10 years after the initial MV infection [4–8]. SSPE is rare, but recent estimated incidences range from 22 to 30–59 per 100,000 in children who acquire measles before the age of 5 years [9–11]. Because the SSPE burden reflects the epidemiology of natural MV infection, an increased incidence of measles may be indicative of a future increase in the number of SSPE cases.

MV, a member of the genus *Morbillivirus* in the *Paramyxoviridae* family of the *Mononegavirales* order, contains a single-stranded, non-segmented, negative-sense RNA genome composed of six genes encoding eight proteins. The phospho (P) and large (L) proteins form the RNA-dependent RNA polymerase (RdRp) complex, which acts as a viral transcriptase or replicase by binding to the RNA genome encapsidated by the nucleocapsid (N) protein; the result is the RNase-resistant ribonucleoprotein (RNP) complex. The P gene encodes two non-structural proteins: V and C [12–14]. The matrix (M) protein facilitates the assembly of the RNP and two envelope glycoproteins [hemagglutinin (H) and the fusion (F) protein], leading to viral particle budding [14,15]. The H and F proteins form an H/F protein complex, which functions in a coordinated manner as fusion machinery [16]. H protein binding to a receptor induces a conformational change in the F protein, triggering viral envelope fusion with the plasma membrane to introduce the RNP into the host cell and initiate infection [17–22]. The H and F proteins, expressed on the surface of infected cells, also support the fusion of the plasma membrane to the membranes of adjacent cells to form multinuclear giant cells (i.e., syncytia), thereby spreading the infection via cell-to-cell fusion [13,23].

MVs infect immune and epithelial cells using signaling lymphocyte activation molecule (SLAM, also known as CD150) and nectin 4 as respective receptors [24–30]. Very rarely, MV invades the CNS where it persists independently of SLAM and nectin 4; neither receptor is expressed in the human CNS [31,32]. This can cause the rare and fatal neurodegenerative disease SSPE several years after recovery from primary acute measles [13], although it is unclear how MV establishes a chronic persistent CNS infection. MV variants obtained from the brains of SSPE patients (SSPE viruses) differ from wild-type MV and have properties such as lack of infectious viral particle production, enhanced cell-to-cell fusion ability, and neuropathogenicity in rodents [33–37]. SSPE viruses harbor characteristic mutations throughout their genomes, particularly in the F and M genes, during infection of the brain [38–45]. Notably, mutations in the ectodomain of the F protein lead to F protein-specific hyperfusogenicity, which is associated with viral spread by cell-to-cell fusion in cells that do not express MV receptors (SLAM and nectin 4); such cells include human neurons [46–48]. This F protein-mediated viral hyperfusogenic phenotype causes the neuropathogenicity exhibited by SSPE viruses [49–52]. Contrarily, recombinant MVs bearing the hypermutant M protein derived from SSPE viruses are not neuropathogenic in rodents [49,53].

The SSPE virus Kobe-1 strain carries viral elements responsible for reducing pathogenicity. The neurovirulence of the Kobe-1 strain was reportedly lower than the neurovirulence of a chimeric recombinant MV possessing the M, F and H genes of the Kobe-1 strain, along with the N, P, and L genes of the wild-type MV [54]. This finding implicated N, P, and L protein-mediated viral RdRp activity in the attenuation of neuropathogenicity. To our knowledge, there is no available information concerning the roles of mutations that restrict the neuropathogenicity of SSPE viruses during the course of SSPE development. Here, we generated chimeric viruses by exchanging the N, P, and L genes of the MV ICB strain and the SSPE virus Kobe-1 strain. Replacement of the L and P genes of MV with the corresponding genes of Kobe-1 restricted viral propagation in neuronal cells by reducing RdRp activity. By evaluating the effect of each amino acid mutation in the L and P proteins on RdRp activity, we identified a potential role for the suppression of RdRp activity in the establishment and/or maintenance of persistent infection in the brain during the transformation of MV into SSPE virus before appearance of clinical signs.

## Results

### The L gene and N and/or P genes of the SSPE virus Kobe-1 strain (SSPEV-L gene and SSPEV-N and/or SSPEV-P genes) did not promote but rather attenuated neuropathogenicity

**Fig 1:**
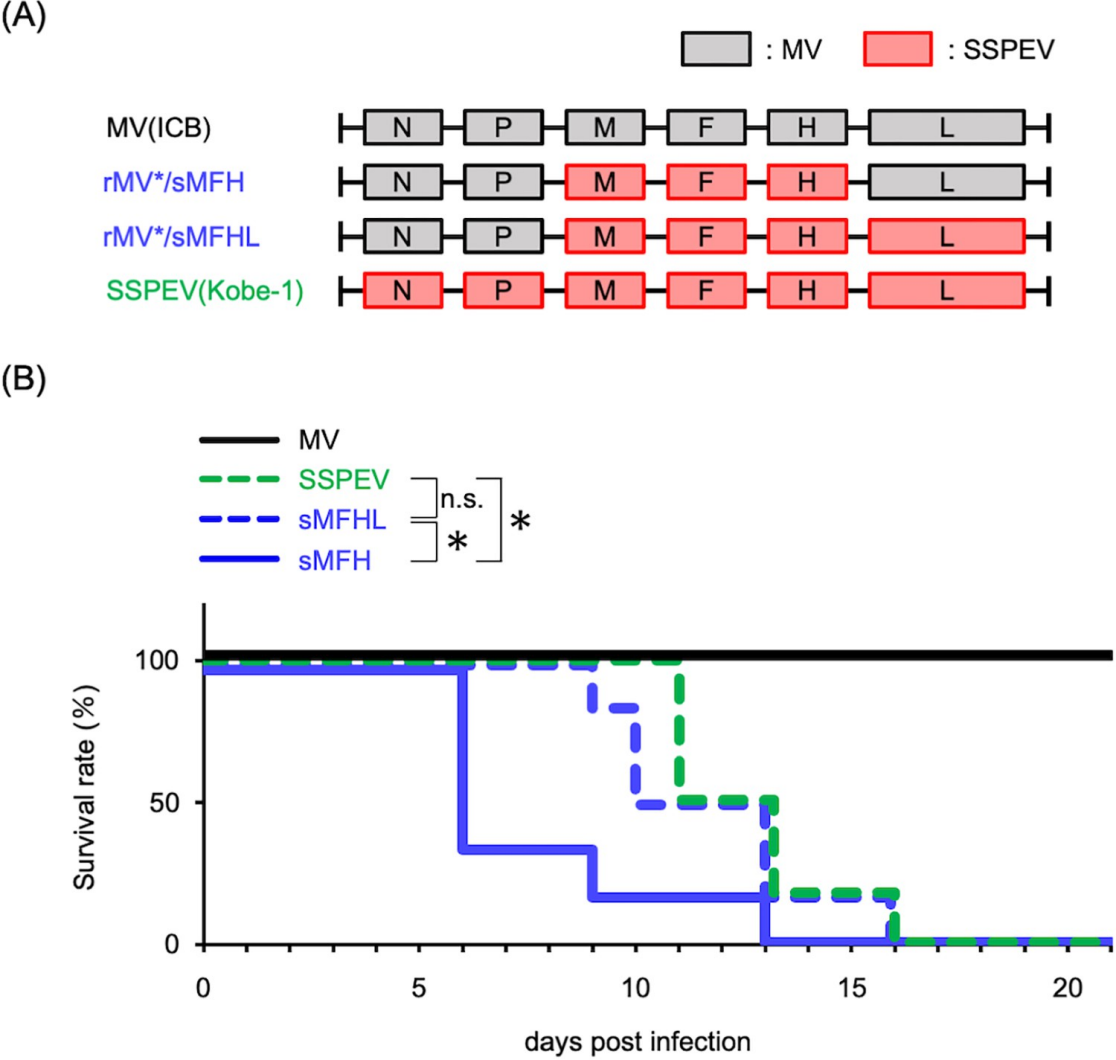
Recombinant MV (rMV) harboring the L gene of the SSPE virus Kobe-1 strain (SSPEV-L gene) attenuated neurovirulence in suckling mice. (**A**) Schematic of the genomes of recombinant chimeric viruses (rMV*). Protein-coding regions derived from the MV ICB strain [MV(ICB)] are shown as gray boxes, whereas protein-coding regions derived from the SSPE virus Kobe-1 strain [SSPEV(Kobe-1)] are shown as red boxes. (**B**) Mortality of suckling mice intracerebrally inoculated with the viruses in (A). Six suckling mice were infected with 7 × 10^2^ PFU of virus and monitored for 21 days. For the p-value calculations, log-rank tests were performed using the survival package in R. If a p-value was less than 0.05, the difference between the survival curves was considered statistically significant. Log-rank tests. *, P < 0.05; n.s., not significant.

By generating enhanced green fluorescent protein (EGFP)-expressing chimeric recombinant MVs of the ICB strain (rMVs), we demonstrated that the rMV harboring the M, F, and H genes of the SSPE virus Kobe-1 strain was more virulent than the Kobe-1 strain in mice when inoculated in the brain [54]. This suggested that the N, P, and/or L genes of the Kobe-1 attenuate neuropathogenicity. To confirm the observation and identify the responsible gene(s), we introduced the L gene of Kobe-1 (SSPEV-L gene) into rMV bearing the M, F, and H genes of Kobe-1 (rMV*/sMFH) and generated rMV*/sMFHL (Fig 1A). As shown in Fig 1B, although rMV*/sMFH was highly lethal in suckling mice that were inoculated intracerebrally, mice inoculated with rMV*/sMFHL died significantly more slowly than mice inoculated with rMV*/sMFH; mice inoculated with SSPE virus Kobe-1 strain [SSPEV(Kobe-1)] died even more slowly than those inoculated with rMV*/sMFHL although statistically not significant. Therefore, we concluded that the SSPEV-L gene attenuates the neurovirulence of chimeric MV in mice, and the N and/or P genes of the SSPE virus Kobe-1 strain (SSPEV-N and/or SSPEV-P genes) may decrease neuropathogenicity.

### The SSPEV-L gene and the SSPEV-N and/or SSPEV-P genes suppressed cell-to-cell fusion and restricted viral propagation in human neuronal cells

**Fig 2:**
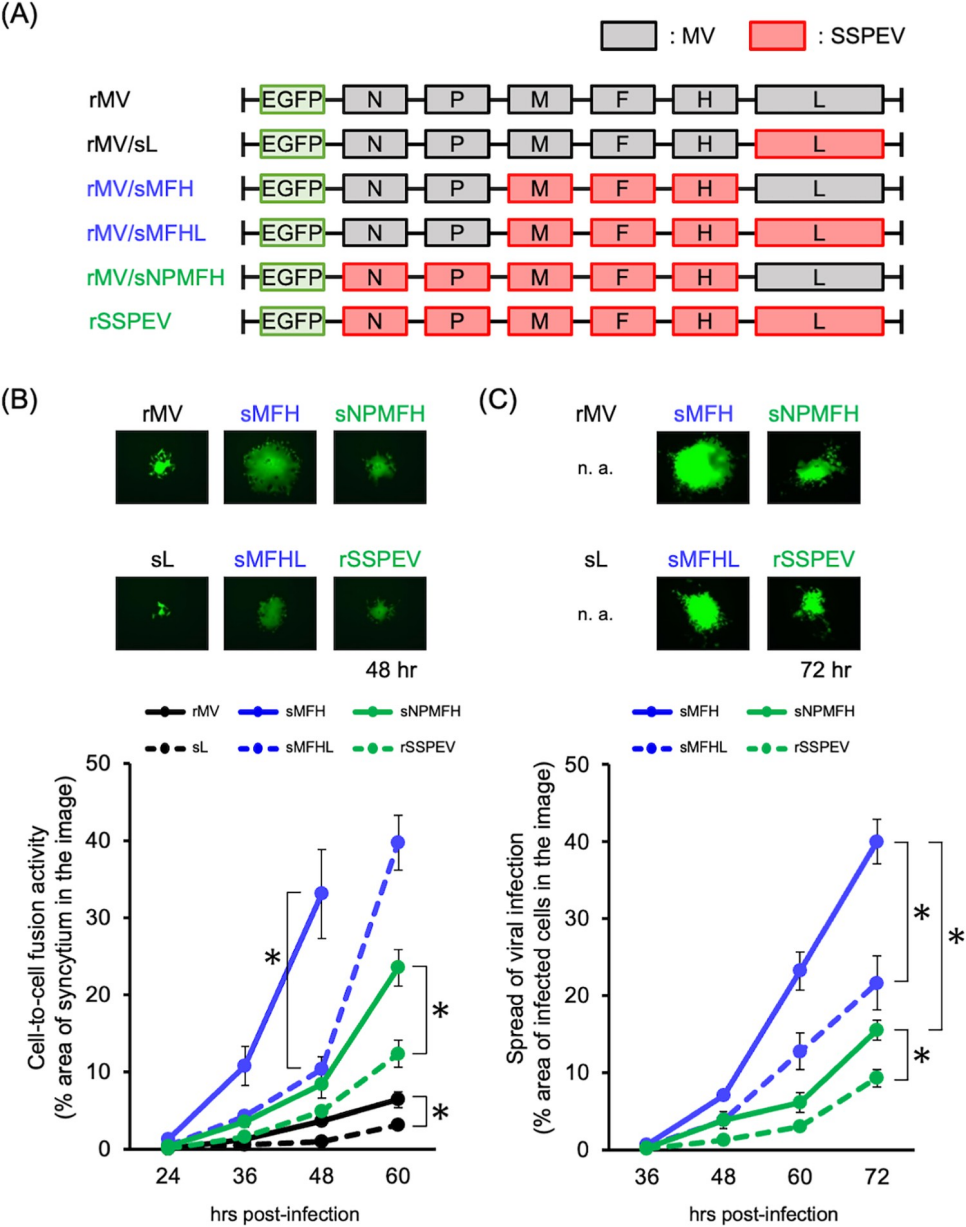
The SSPEV-L gene and the SSPEV-N and/or SSPEV-P genes suppressed cell-to-cell fusion and propagation of rMVs in neuronal cells. (**A**) Schematic of the genomes of EGFP-expressing rMVs. Colors of protein-coding regions correspond to Fig 1A. (**B**) Cell-to-cell fusion of rMVs in Vero/hSLAM cells. Vero/hSLAM cells were infected with EGFP-expressing cell-free rMVs, and syncytia derived from individual infected cells were observed for 60 h under a fluorescence microscope. Representative photographs of syncytia at 48 h post-infection are shown (upper panel). Magnification, ×200. Time course of syncytia enlargement shown as the percentage area of EGFP-positive syncytia, calculated using ImageJ (lower panel). Representative photographs at each time point are presented in S1A Fig. Data from five images are shown as means ± standard deviations. sL, rMV/sL; sMFH, rMV/sMFH; sMFHL, rMV/sMFHL; sNPMFH, rMV/sNPMFH. Unpaired Student’s t-test. *, P < 0.05; n.s., not significant. (**C**) Propagation of rMVs in human neuronal cells. SH-SY5Y cells were infected with EGFP-expressing cell-free rMVs, and the spread of viral infection derived from a single infected cell was observed for 72 h under a fluorescence microscope. Representative photographs of infected cells at 72 h post-infection are shown (upper panel). Magnification, ×200. Time course of infection expansion shown as the percentage area of EGFP-positive cells, calculated using ImageJ (lower panel). Photographs at each time point are presented in S1B Fig. Data from five images are shown as means ± standard deviations. sMFH, rMV/sMFH; sMFHL, rMV/sMFHL; sNPMFH, rMV/sNPMFH. Unpaired Student’s t-test. *, P < 0.05; n.s., not significant.

The neuropathogenicity of SSPE viruses is associated with viral hyperfusogenicity, which enables cell-to-cell spread of infection among neuronal cells lacking authentic viral receptors [49–52,54]. Although viral hyperfusogenicity is regulated by the fusion activity of the F protein [5,48,55], we examined the role of the L gene by introducing the SSPEV-L gene into EGFP-expressing rMV (rMV) and into rMV bearing the M, F and H genes of the Kobe-1 (rMV/sMFH); alternatively, we replaced the L gene of the EGFP-expressing recombinant SSPE virus Kobe-1 strain (rSSPEV) with the L gene of the MV ICB strain (rMV/sNPMFH) (Fig 2A). When inoculated into Vero/hSLAM cells, rMV/sL, rMV/sMFHL, and rSSPEV harboring the SSPEV-L gene demonstrated weaker cell-to-cell fusion ability compared with rMV, rMV/sMFH, and rMV/sNPMFH, respectively (Fig 2B). Next, we used these viruses to inoculate SH-SY5Y human neuronal cells. As shown in Fig 2C, infections of rMV/sMFHL and rSSPEV spread more slowly than infections of rMV/sMFH and rMV/sNPMFH, respectively. Therefore, the SSPEV-L gene restricted viral growth by suppressing cell-to-cell spread among neuronal cells, reducing neuropathogenicity in mice (Fig 1B). Comparisons of rMV/sMFH and rMV/sNPMFH, or rMV/sMFHL and rSSPEV, revealed that rMV/sNPMFH and rSSPEV carrying the SSPEV-N and SSPEV-P genes showed less efficient cell-to-cell fusion and viral growth in neuronal cells, indicating that the SSPEV-N and/or SSPEV-P genes have suppressive effects. rMV and rMV/sL bearing the M, F, and H genes of the MV ICB strain did not spread among neuronal cells. These findings confirmed that the principal growth determinants of rMVs in neuronal cells lacking MV receptors are the F and M genes of Kobe-1 (i.e., SSPEV-F and SSPEV-M genes) as previously proved [54], which may be modified by the L gene and the N and/or P genes.

### The SSPEV-L gene and the SSPEV-N and/or SSPEV-P genes restricted cell-surface expression of the F protein

**Fig 3:**
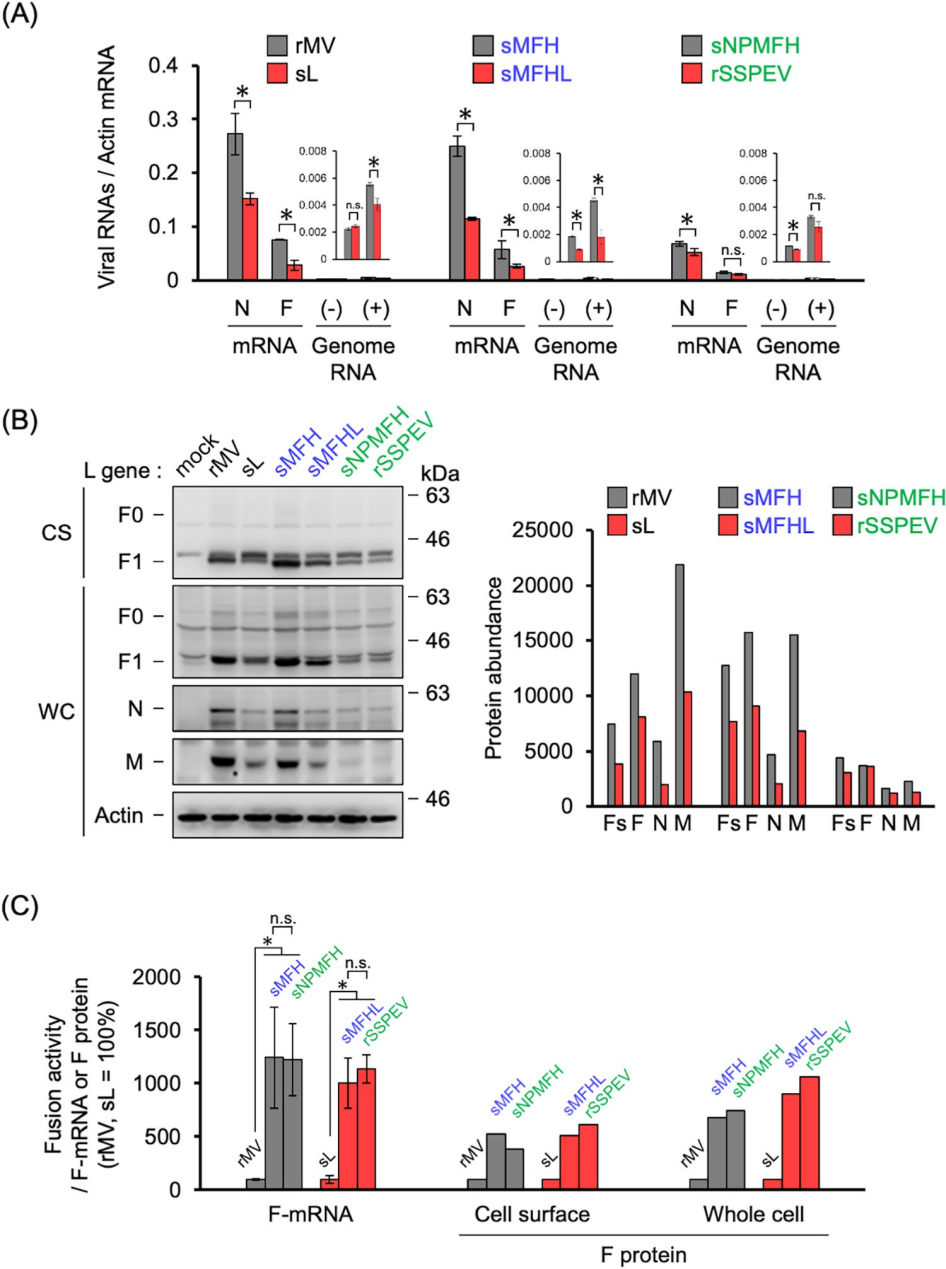
rMVs carrying the SSPEV-L gene and the SSPEV-N and/or SSPEV-P genes reduced viral RNA production and restricted cell-surface expression of the F protein. (**A**) Viral RNA levels in cells infected with rMVs. Vero/hSLAM cells were infected with the rMVs depicted in Fig 2A and incubated at 37°C for 48 h in the presence of fusion-inhibiting peptide. Total RNA extraction, reverse transcription, and quantitative PCR were performed. Data from three independent experiments are shown as means ± standard deviations. (-), negative-sense genome; (+), positive-sense genome. Unpaired Student’s t-test. *, P < 0.05; n.s., not significant. (**B**) F protein expression at the surface of cells infected with rMVs. Vero/hSLAM cells were infected with the rMVs in Fig 2A. Cell-surface proteins were biotinylated to detect F protein before preparation of whole-cell lysates. Samples were subjected to immunoblot analysis using anti-MV F, anti-MV N, anti-MV M, and anti-β-actin primary antibodies. A representative image of several experiments is shown (left panel). Inactive F0 and active F1 forms of the F protein are shown; molecular markers are indicated on right. CS, cell-surface fraction; WC, whole-cell fraction. Intensities of F1, N, and M protein bands were quantified using ImageJ (right panel). Fs, F1 protein in CS; F, F1 protein in WC. (**C**) Specific viral cell-to-cell fusion activity relative to F mRNA and F1 protein levels. Specific cell-to-cell fusion activity was calculated through division of cell-to-cell fusion at 48 h post-infection in Fig 2B by the F mRNA or F1 protein level in Fig 3A and 3B. The value of rMV or rMV/sL (sL) was set at 100%. Unpaired Student’s t-test. *, P < 0.05; n.s., not significant.

The N, P, and L proteins (encoded by the N, P, and L genes, respectively) form the RNP complex for transcription of viral mRNA and replication of the viral genome. The restricted growth of rMVs harboring the SSPEV-L gene and the SSPEV-N and/or SSPEV-P genes in neuronal cells may be caused by altered RdRp activity, thereby affecting F-protein expression at the cell surface. As shown in Fig 3A, the F mRNA levels were lower in cells infected with rMV/sL, rMV/sMFHL, or rSSPEV than in cells infected with rMV, rMV/sMFH, or rMV/sNPMFH, respectively [i.e., the corresponding rMVs bearing the L gene of the MV ICB strain (MV-L gene)]. Thus, the surface F protein levels were significantly reduced in cells infected with rMV/sL, rMV/sMFHL, or rSSPEV (Fig 3B). When compared between rMV/sMFH and rMV/sNPMFH, or rMV/sMFHL and rSSPEV, the F mRNA and protein levels were lower in cells infected with rMV/sNPMFH or rSSPEV carrying the SSPEV-N and SSPEV-P genes. The reductions of N mRNA, viral genome (+ and–senses), and F mRNA suggested that the SSPEV-L protein and the SSPEV-N and/or SSPEV-P proteins suppressed the RdRp activity of the RNP complex, thereby decreasing cell-surface F-protein expression.

We next examined the effects of the SSPEV-L protein and the SSPEV-N and/or SSPEV-P proteins on viral cell-to-cell fusion. Relative fusion activity was calculated through division of the cell-to-cell fusion activity (Fig 2B) by the F mRNA or protein level (Fig 3A or 3B). As shown in Fig 3C, whereas the relative fusion activities of rMV and rMV/sL bearing the MV-M, MV-F, and MV-H genes were very low, the relative fusion activities of rMV/sMFH, rMV/sMFHL, rMV/sNPMFH, and rSSPEV carrying the SSPEV-M, SSPEV-F, and SSPEV-H genes were high. These differences were presumably related to the enhanced fusogenicity of the SSPEV-F protein, under the cooperative assistance by the SSPEV-M protein [54]. The relative cell-to-cell fusion activities of rMV/sMFH, rMV/sMFHL, rMV/sNPMFH, and rSSPEV were not significantly different, suggesting that the SSPEV-L protein and the SSPEV-N and/or SSPEV-P proteins did not directly influence cell-to-cell fusion. The suppressed cell-to-cell fusion activities of rMV/sL, rMV/sMFHL, and rSSPEV compared with rMV, rMV/sMFH, and rMV/sNPMFH, respectively (Fig 2B), were related to the decreased cell-surface F protein level that was caused by the suppression of RdRp activity.

These results indicated that cell-to-cell fusion of rMVs is principally regulated by the fusogenicity of the F protein, which may be modulated by the F protein level that is determined by the RdRp activity of the viral RNP complex.

### SSPEV-L and SSPEV-P proteins suppressed RdRp activity

**Fig 4:**
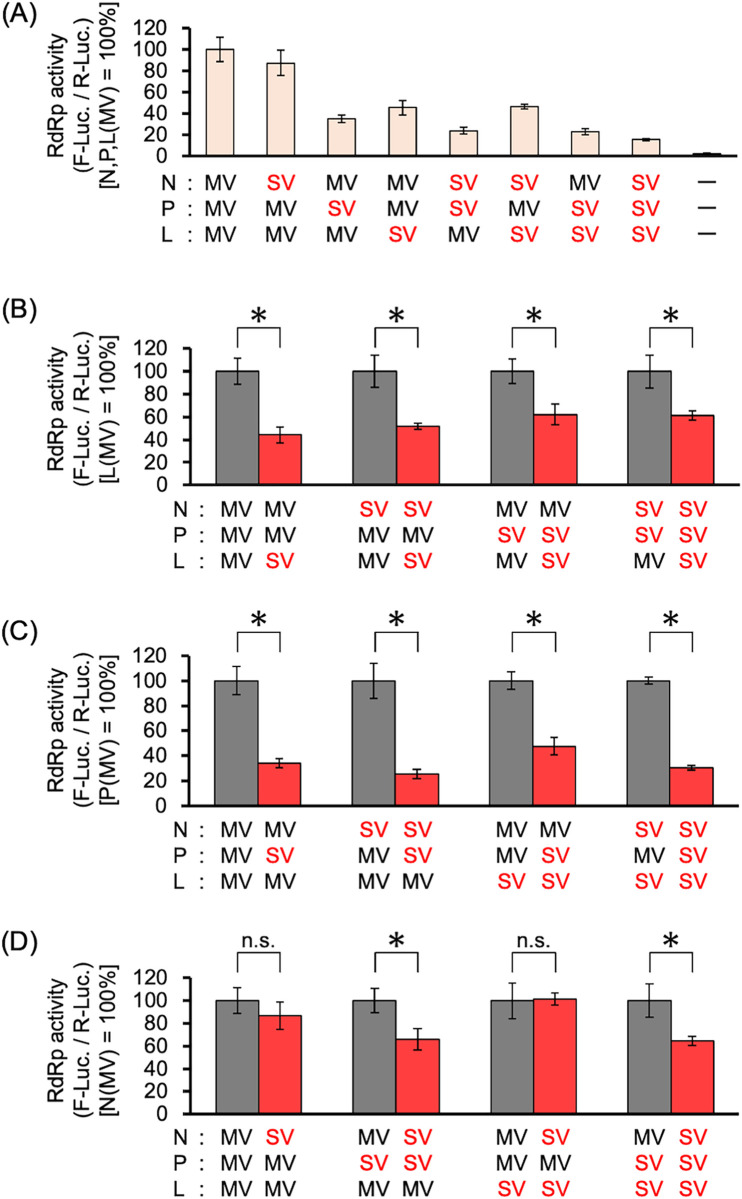
SSPEV-L and SSPEV-P proteins decrease RdRp activity. (**A**) RdRp activities of the N, P, and L proteins of the MV ICB strain (MV) or SSPE virus Kobe-1 strain (SV) in various combinations. Vero/hSLAM cells were transfected with plasmids expressing the N, P, and L proteins of MV or SV in combination, together with the MV mini-genome plasmid encoding the firefly luciferase gene and the *Renilla* luciferase-expressing plasmid. After incubation at 37°C for 24 h, RdRp activity was determined. Data from three independent experiments are shown as means ± standard deviations. (**B**) Relative RdRp activities for evaluation of the effect of the L protein. RdRp activities in (A) were compared between the MV-L protein (100%) and the SV-L protein with the same combinations of N and P proteins. Unpaired Student’s *t*-test. *, P < 0.05. (**C**) Relative RdRp activities for evaluation of the effect of the P protein. RdRp activities in (A) were compared between the MV-P protein (100%) and the SV-P protein with the same combinations of N and L proteins. Unpaired Student’s *t*-test. *, P < 0.05. (**D**) Relative RdRp activities for evaluation of the effect of the N protein. RdRp activities in (A) were compared between the MV-N protein (100%) and the SV-N protein with the same combinations of P and L proteins. Unpaired Student’s *t*-test. *, P < 0.05; n.s., not significant.

We analyzed the RdRp activity of the RNP complex composed of the N, P, and L proteins from the MV ICB strain or the SSPE virus Kobe-1 strain in various combinations (Fig 4A). Mini-genome assays showed that the SSPEV-L and SSPEV-P proteins strongly suppressed RdRp activity (Fig 4B and 4C), but the SSPEV-N protein moderately reduced RdRp activity in the presence of the SSPEV-P protein (Fig 4D). There may be differences between the MV-P protein and the SSPEV-P protein in terms of their interactions with the N protein. When the MV-P gene of rMV/sMFH or rMV/sMFHL was replaced with the SSPEV-P gene (S2A Fig), the resulting rMV/sPMFH or rMV/sPMFHL exhibited substantial suppression of viral cell-to-cell fusion ability and growth in neuronal cells. In contrast, replacement of the MV-N gene with the SSPEV-N gene did not lead to a change in viral characteristics (S2B and S2C Fig).

The results indicated that the suppressed propagation in neuronal cells and reduced cell-to-cell fusion of rMV/sNPMFH and rSSPEV bearing the SSPEV-N and SSPEV-P genes, compared with rMV/sMFH and rMV/sMFHL bearing the MV-P and MV-N genes (Fig 2), is caused by the reduction of RdRp activity (Fig 3A) in a manner mediated by the SSPEV-P protein, rather than the SSPEV-N protein.

### Three amino acid mutations in the SSPEV-L protein sharply suppressed RdRp activity

To identify mutations involved in the reduction of RdRp activity, mutations in the SSPEV-P and SSPEV-L proteins were sequentially introduced into the MV-P and MV-L proteins, respectively; the effects were evaluated by mini-genome assays.

**Fig 5:**
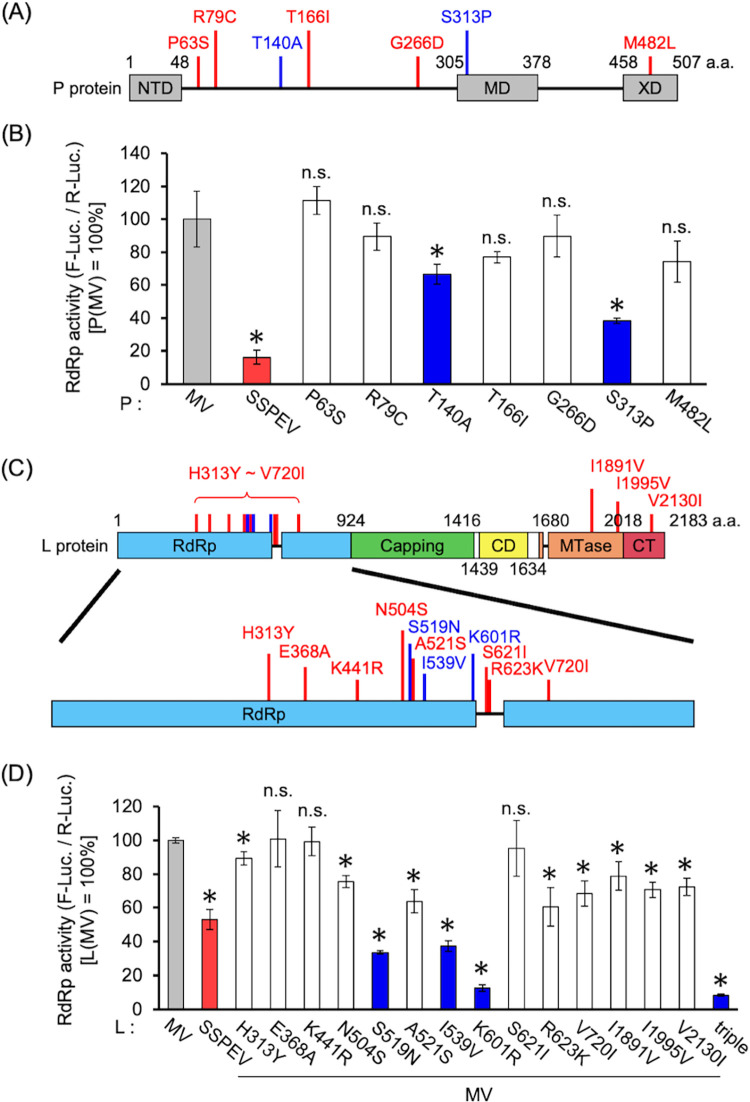
Multiple amino acid mutations in the P and L proteins reduced RdRp activity. (**A**) Schematic of the locations of amino acid mutations in the SSPEV-P protein. NTD, N-terminal domain; MD, multimerization domain; XD, X domain. (**B**) Effects of the mutations of SSPEV-P protein on RdRp activity. Vero/hSLAM cells were transfected with a plasmid expressing the P protein with each mutation, together with plasmids expressing the N and L proteins of MV, an MV mini-genome plasmid encoding the firefly luciferase gene, and the *Renilla* luciferase-expressing plasmid. RdRp activity was determined as in Fig 4A. Data from three independent experiments are shown as means ± standard deviations. Unpaired Student’s *t*-test. *, P < 0.05 compared with MV-P protein; n.s., not significant compared with MV-P protein. (**C**) Schematic of the locations of amino acid mutations in the SSPEV-L protein. RdRp, RdRp domain; Capping, capping domain; CD, connecting domain; MTase, methyltransferase domain; CT, C-terminal domain. (**D**) Effects of mutations of the SSPEV-L protein on RdRp activity. Vero/hSLAM cells were transfected with a plasmid expressing the L protein with each mutation, together with plasmids expressing the N and P proteins of MV, an MV mini-genome plasmid encoding the firefly luciferase gene, and the *Renilla* luciferase-expressing plasmid. RdRp activity was determined as in Fig 4A. Triple, L protein carrying S519N, I539V, and K601R mutations. Data from three independent experiments are shown as means ± standard deviations. Unpaired Student’s t-test. *, *P* < 0.05 compared with MV-L protein; n.s., not significant compared with MV-L protein.

Mutations were located throughout the SSPEV-P protein. Among them, T140A and S313P significantly reduced RdRp activity; MV-P proteins with single point mutations other than T140A and S313P exhibited non-significant reductions of RdRp activity (Fig 5B). The RdRp activity of the SSPE virus Kobe-1 strain may have decreased through the accumulation of mutations in the P protein. Amino acid 313 is located in the multimerization domain (MD) (Fig 5A), and S313P loosens the α-helix of the MD, thereby decreasing RdRp activity [56].

Amino acid mutations in the SSPEV-L protein were located in the RdRp, methyltransferase (MTase), and C-terminal (CT) domains (Fig 5C). S519N, I539V, and K601R substantially decreased the RdRp activity of the MV-L protein to a level below the activity of the SSPEV-L protein (Fig 5D). These mutations were around the site of RdRp activity in the RdRp domain, suggesting a direct relationship with RNA synthesis. Indeed, the triple-mutant MV-L protein possessing S519N, I539V, and K601R [L(triple) protein] exhibited extremely low RdRp activity.

### Suppression of RdRp activity restricted progeny virus production and may promote persistent infection

The suppression of RdRp activity by the SSPEV-L and SSPEV-P proteins suppressed viral cell-to-cell spread among neuronal cells. Because the onset and/or aggravation of SSPE are caused by and correlated with the propagation of SSPE viruses in the brain, reduced RdRp activity presumably did not contribute to these events. Although it is impossible to isolate and examine virus persisting in the brain of an SSPE patient before the appearance of clinical signs, we suspect that MV suppresses the expression of viral proteins and the release of viral particles, thus establishing a persistent infection. This suppression may involve reduced RdRp activity.

**Fig 6:**
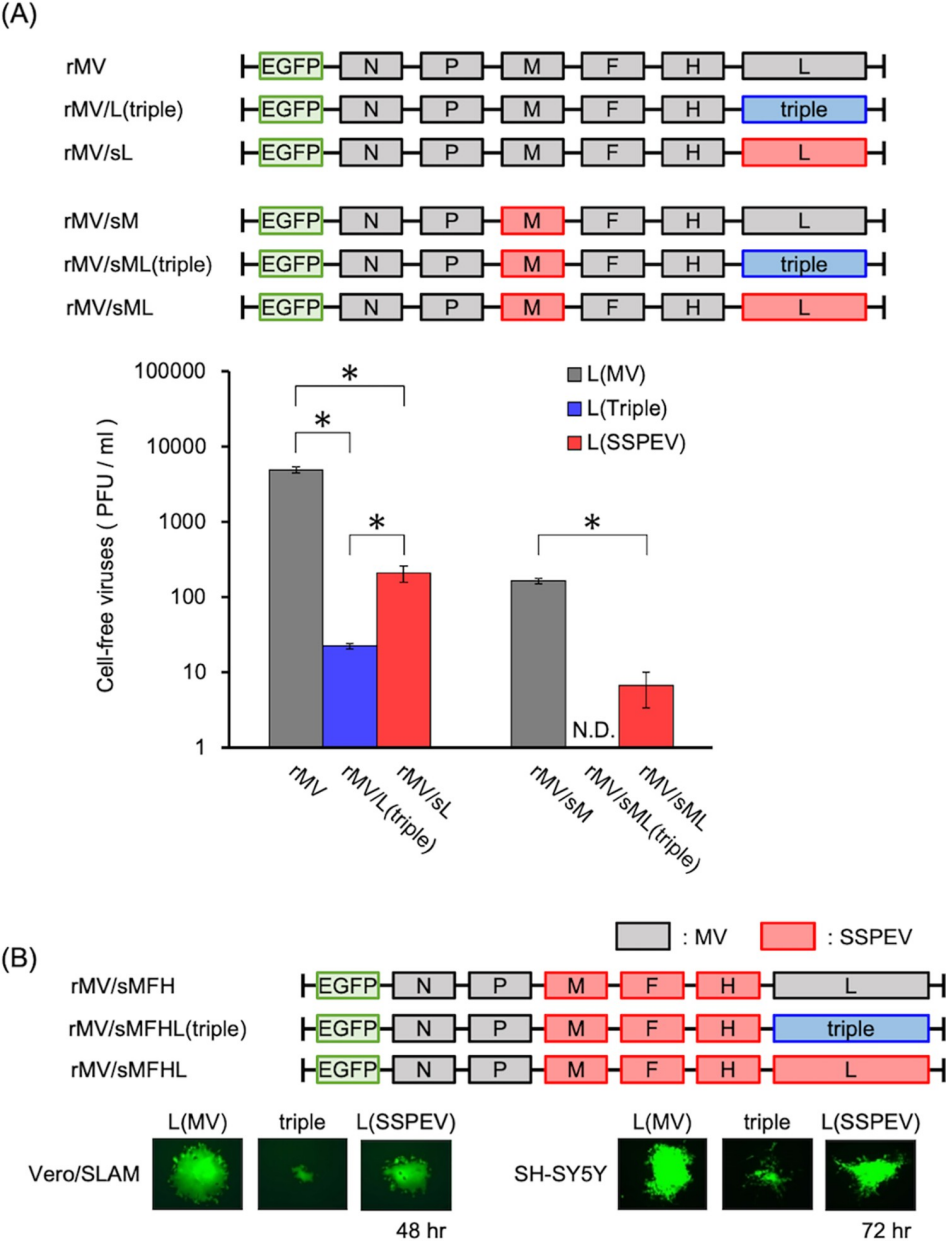
The triple-mutant L protein [L(triple) protein] limited progeny virus production and restricted viral propagation in neuronal cells. (**A**) Cell-free infectious viruses released from cells infected with rMVs. Schematic of the genomes of the rMVs is shown (upper panel). Colors of the protein-coding regions derived from MV or SSPEV correspond to Fig 1A. Triple (blue box) represents the MV-L gene harboring S519N, I539V, and K601R mutations. Vero/hSLAM cells were infected with rMVs and incubated at 37°C for 4 days; the medium was changed every 24 h. After centrifugation, cell-free viruses in culture medium were titrated in Vero/hSLAM cells. Infectious virus titers at day 4 post-infection are shown. Titers over 4 days are presented in S3 Fig. Data from three independent experiments are shown as means ± standard deviations. Unpaired Student’s *t*-test. *, P < 0.05; N.D., not detected. (**B**) Effects of the L(triple) protein on viral cell-to-cell fusion and propagation in neuronal cells. Schematic of the genomes of rMVs bearing the SSPEV-M, SSPEV-F, and SSPEV-H genes is shown (upper panel). Colors of the protein-coding regions are as in (A). Vero/hSLAM (left panel) and SH-SY5H cells (right panel) were infected with rMVs, incubated at 37°C, and monitored under a fluorescence microscope. Representative photographs derived from a single infected cell were acquired at 48 and 72 h. Magnification, ×200.

Next, we evaluated the effect of L protein with S519N, I539V, and K601R mutations [L(triple) protein] on progeny virus production and growth in neuronal cells. As shown in Fig 6A, rMV carrying the SSPEV-L protein (rMV/sL) and the L(triple) protein [rMV/L(triple)] produced < 10% and < 1% infectious rMV, respectively. Lack of progeny virus release, typical of SSPE viruses, is caused by mutations in the M protein [49,54], but replacement of the MV-M gene with the SSPEV-M gene did not abolish virus particle production (see rMV/sM in Fig 6A) [34]. The reduction of RdRp activity restricted virus release, and rMV/sML(triple) carrying the SSPEV-M protein and the L(triple) protein lost the ability to release infectious virus. Because the SSPEV-F and SSPEV-M proteins are indispensable for propagation in neuronal cells [54], we next evaluated the effect of the triple mutant on viral cell-to-cell fusion and growth in neuronal cells using rMVs harboring the SSPEV-M, SSPEV-F, and SSPEV-H genes. The triple mutants strongly suppressed cell-to-cell fusion of rMV/sMFHL(triple) and limited viral spread in SH-SY5Y neuronal cells, compared with rMV/sMFH and rMV/sMFHL (Fig 6B).

Therefore, the suppression of RdRp activity restricted viral particle release and cell-to-cell spread among neuronal cells, suggesting that the L(triple) protein (which had substantially reduced RdRp activity) promoted the establishment of persistent infection in the brain by limiting viral propagation.

### Suppression of RdRp activity by the L(triple) protein was restored by induction of other mutations in the SSPEV-L protein

**Fig 7:**
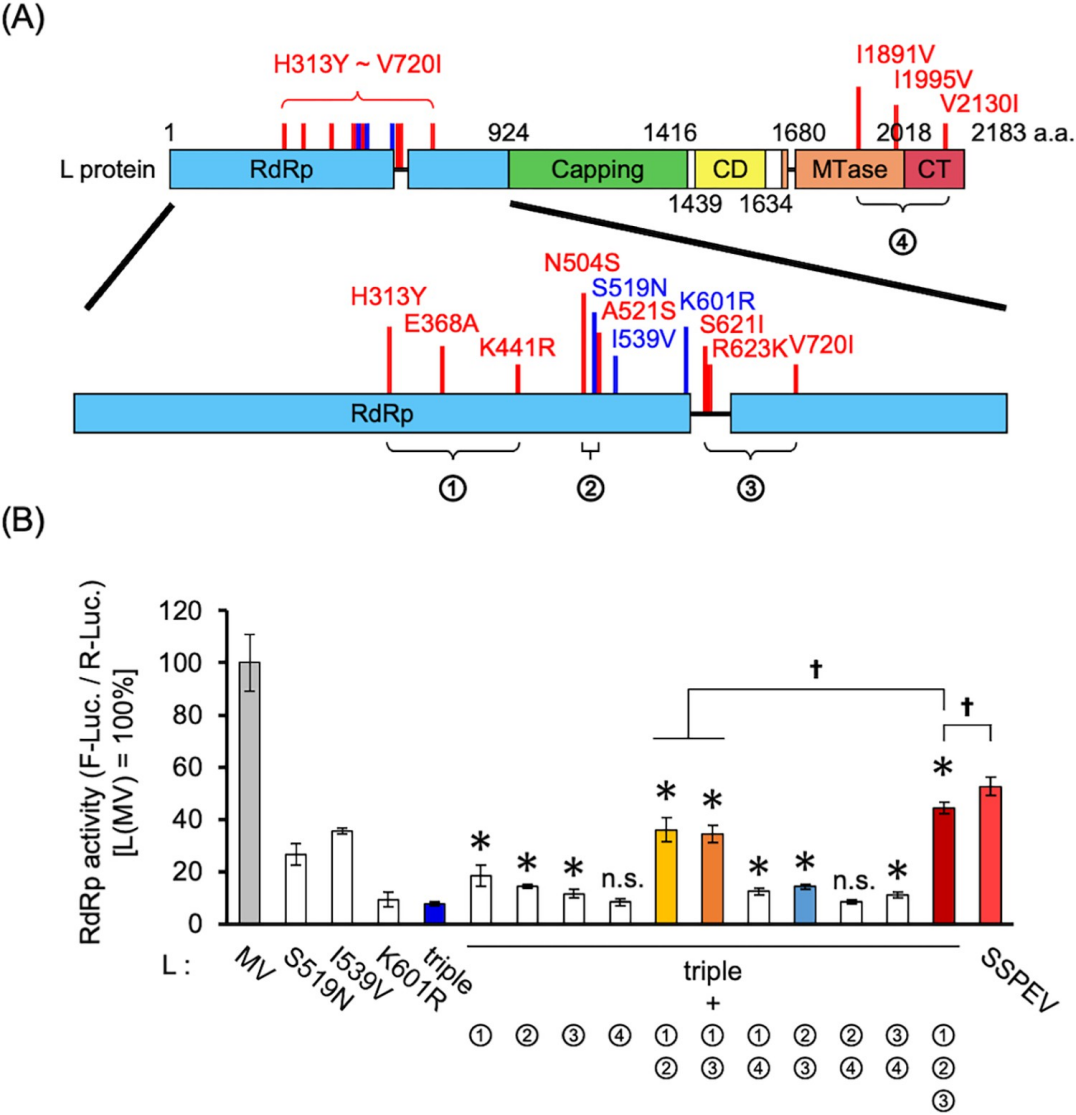
Reduced RdRp activity of the L(triple) protein was restored by introduction of other mutations into the SSPEV-L protein. (**A**) Schematic of the locations of amino acid mutations in the SSPEV-L protein. S519N, I539V, and K601R mutations are shown in blue. Other mutations (red): group 1 (H313Y, E368A, and K441R), group 2 (N504S and A521S), group 3 (S621I, R623K, and V720I), and group 4 (I1891V, I1995V, and V2130I). (**B**) RdRp activities of the L(triple) protein in combination with groups 1, 2, 3, and 4 mutations. Amino acid substitutions are listed in S1 Table. RdRp activities were measured as in Fig 5. Data from three independent experiments are shown as means ± standard deviations. Unpaired Student’s *t*-test. *, P < 0.05 compared with triple-mutant L protein; n.s., not significant compared with triple-mutant L protein; †, P < 0.05.

The L(triple) protein promoted persistent infection at the stage before onset of SSPE. To examine the effects of other mutations in the SSPEV-L protein on the RdRp activity of the L(triple) protein, we introduced mutations into the L(triple) protein (Fig 7A). Mutations in groups 1, 2, and 3 (in the RdRp domain) significantly increased the RdRp activity of the L(triple) protein, which was enhanced by combinations of these mutations (Fig 7B). The substantial enhancements in groups 1+2 and 1+3 demonstrated that the suppressed RdRp activity of the L(triple) protein was restored by mutations in group 1, through synergistic interactions with mutations in group 2 or 3. The addition of all mutations led to an additional increase in RdRp activity, which was further enhanced by the addition of mutations in group 4 (SSPEV-L protein in Fig 7B); however, mutations in group 4 (i.e., MTase and CT domains) alone did not enhance activity. Therefore, the RdRp activity of the L(triple) protein was substantially reduced by S519N, I539V, and K601R mutations; it was restored by the cumulative addition of other mutations.

### Restoration of RdRp activity corresponded to viral propagation in neuronal cells

**Fig 8:**
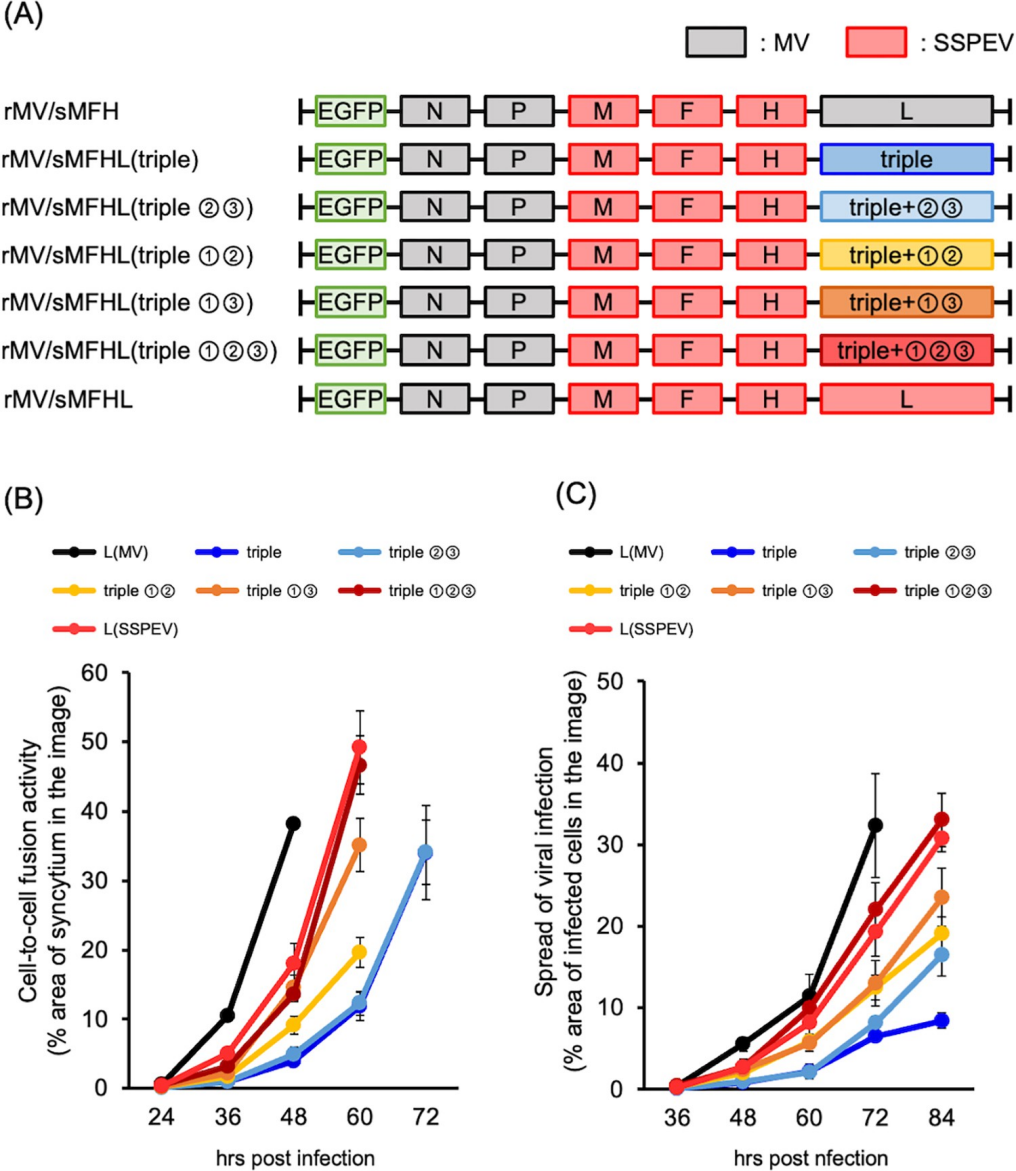
Restoration of RdRp activity was associated with enhancement of cell-to-cell fusion and propagation of rMVs in neuronal cells. (**A**) Schematic of EGFP-expressing rMV/sMFH genomes harboring amino acid substitutions in the L protein in Fig 7. (**B**) Cell-to-cell fusion. Vero/hSLAM cells were infected with the cell-free rMVs in (A) and enlargement of fused cells was monitored under a fluorescence microscope; photographs were obtained at 12-h intervals. Representative photographs are shown in S5A Fig. Cell-to-cell fusion activity was quantified as in Fig 2B. Data from five images are shown as means ± standard deviations. (**C**) Viral propagation in neuronal cells. SH-SY5Y cells were infected with the cell-free rMVs in (A) and the spread of infection from a single infected cell was observed under a fluorescence microscope; photographs were obtained at 12-h intervals. Representative photographs are shown in S5B Fig. Spread of viral infection was determined as in Fig 2C. Data from five images are shown as means ± standard deviations.

To determine whether restoration of the RdRp activity of the L(triple) protein affects viral behavior, we generated rMVs harboring the L(triple) protein with the other mutations (Fig 7) based on rMV/sMFHL(triple) (Fig 8A). The RdRp activity of the L(triple) protein was increased by the addition of the four groups of mutations (Fig 7B); the F mRNA level was increased in cells infected with each virus (S4A Fig), and the level of cell-surface F-protein expression was also increased (S4B Fig). Accordingly, rMVs increased cell-to-cell fusion (Fig 8B) and the spread of infection among neuronal cells (Fig 8C) in a manner that corresponded to RdRp activity. Control virus (rMV/sMFH) bearing the MV-L protein with the highest RdRp activity produced the greatest cell-surface F-protein expression and caused the greatest propagation in neuronal cells. Therefore, RdRp activity corresponds to viral cell-to-cell fusion and regulates viral propagation in neuronal cells if the virus can successfully infect neuronal cells (i.e., it contains SSPEV-F and SSPEV-M proteins). Because growth in neuronal cells is associated with viral neuropathogenicity (Figs 1 and 2) [54], the neurovirulence of rMV/sMFHL(triple) should have been extremely low and increased with the enhancement of RdRp activity in a manner mediated by the accumulation of other mutations in the SSPEV-L protein.

### Effects of mutations in the SSPE virus Kobe-1 strain on SSPE progression

**Fig 9:**
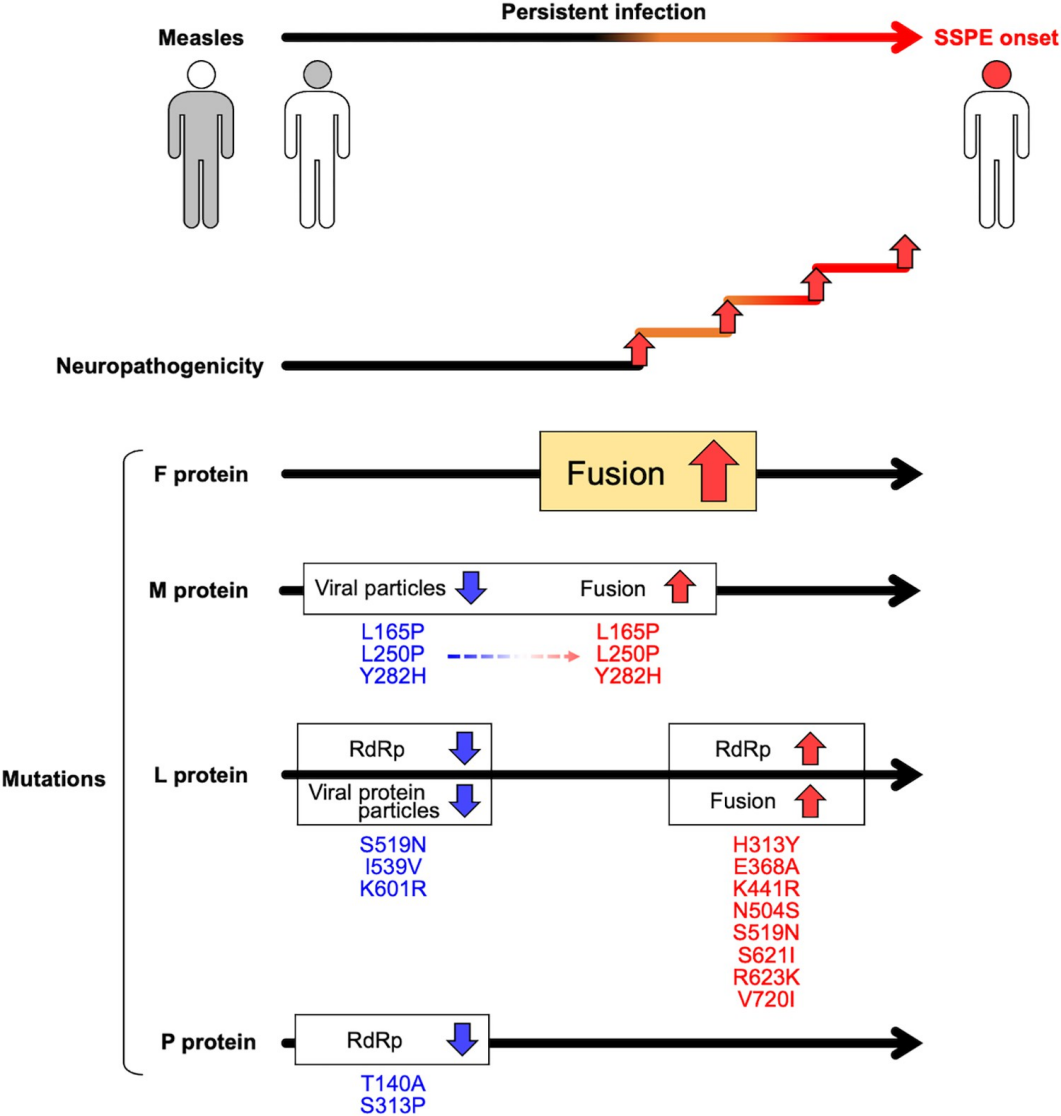
Possible roles of mutations accumulated in the SSPE virus Kobe-1 strain in SSPE. Mutations in blue suppress the functions of viral proteins and may contribute to the establishment and maintenance of persistent infection. Mutations in red enhance viral protein functions to promote viral propagation in the brain, resulting in the onset of SSPE. Mutations in the M protein were described previously [34,54].

Based on our findings, we constructed a model of the contributions of mutations in the SSPE virus Kobe-1 strain to the progression of SSPE (Fig 9). The finding that the SSPEV-L protein attenuated (rather than promoted) viral neuropathogenicity prompted us to consider the role of the L protein in persistent infection before the onset of SSPE. Under the assumption that the tracks of mutations must be present in the genome of an SSPE virus, we analyzed the effects of mutations of the SSPEV-L protein on RdRp activity. RdRp activity was substantially reduced by the presence of S519N, I539V, and K601R mutations, but it was restored by the addition of other mutations (Fig 7). The reduction of RdRp activity could promote persistence by hindering progeny virus production and viral protein expression, thereby mediating immune escape. Two mutations in the SSPEV-P protein that suppressed viral RdRp activity also promoted the establishment and maintenance of persistent infection. However, the restoration of RdRp activity may explain efficient Kobe-1 virus spread in the brain after the acquisition of fusogenic mutations in the F protein. Mutations in the SSPEV-M protein hindered viral particle production (Fig 6A) under the suppressed viral RdRp activity, thereby avoiding host immunity, but enhanced cell-to-cell fusion, thereby promoting viral spread among neuronal cells, and increasing neuropathogenicity under synergistic cooperation with the fusogenic SSPEV-F protein [54]. The accelerated fusion activity of the SSPEV-F protein was indispensable for cell-to-cell spread in the brain, which was enhanced by mutations in the SSPEV-L and SSPEV-M proteins. Neuropathogenicity would presumably increase as mutations accumulated, leading to the onset of SSPE. We are conducting further analyses of mutations in the SSPEV-F protein that are responsible for its fusogenicity.

## Discussion

SSPE is a very rare late complication of MV infection that occurs in apparently healthy children, 7 to 10 years after acute measles [4–8]. Although virus cannot be isolated during the transformation of MV into SSPE virus before the appearance of clinical signs, the development of SSPE may occur as follows. First, MV enters the brain, presumably during the acute exanthematous phase [57]. Next, MV infects neurons that lack receptors for MV. Nectin-elicited cytoplasm transfer, which transports transmembrane and cytoplasmic proteins via cell-to-cell contacts established by the nectin adhesive interface, can spread MV infection from epithelial cells to primary neurons [58]. Within a neuron, MV undergoes mutations to avoid immune recognition [5,59]. The M gene is highly mutated in nearly all SSPE cases, impairing the formation of viral particles and promoting viral escape from neutralizing antibodies [8]. MV reproduces intracellularly in a non-cytopathic manner to avoid destroying host neurons, thereby establishing persistent infection [7]. The mutations and mechanisms involved in this step are unclear. Then the F protein acquires hyperfusogenicity, which facilitates cell-to-cell fusion and transneuronal viral spread in the absence of MV receptors [47]. Cell adhesion molecules 1 and 2 [60,61], or neurokinin-1 [62], may enable MV to induce neuronal fusion. Mutation of the F protein is essential for the advancement from persistent to reproductive infection. When clinical signs of neurological disease occur (e.g., behavioral changes, decreased school performance, and/or seizures), SSPE virus is widely distributed in neurons of the CNS [8,11]. The inflammatory response in the brain to persistent SSPE virus leads to extensive tissue damage and cerebral atrophy. Clinically, SSPE is characterized by florid panencephalitis [7,8].

The role of the RdRp activity of the L and P proteins in SSPE development is unclear. Cattaneo *et al*. [63] reported that transcription decreases at each gene junction in the MV genome in the brains of SSPE patients, presumably reducing the expression of viral envelope proteins on the surface of brain cells. This phenomenon may explain the lack of viral budding and the ability to escape from immune surveillance, thereby enabling MV persistent infection. Notably, they presumed that the phenomena were brain-specific based on several host factors and did not evaluate mutations in the L and/or P proteins [64]. In the present study, we showed that suppression of the RdRp activity by the SSPEV-L and SSPEV-P proteins decreased the expression levels of viral proteins, enabling the establishment of persistent infection in the brain during the incubation period. The virus could then evade host immunity and suppress neuronal destruction by avoiding excessive propagation. The reduction of RdRp activity may be necessary to establish and/or maintain persistent MV infection in the brain.

**Fig 10:**
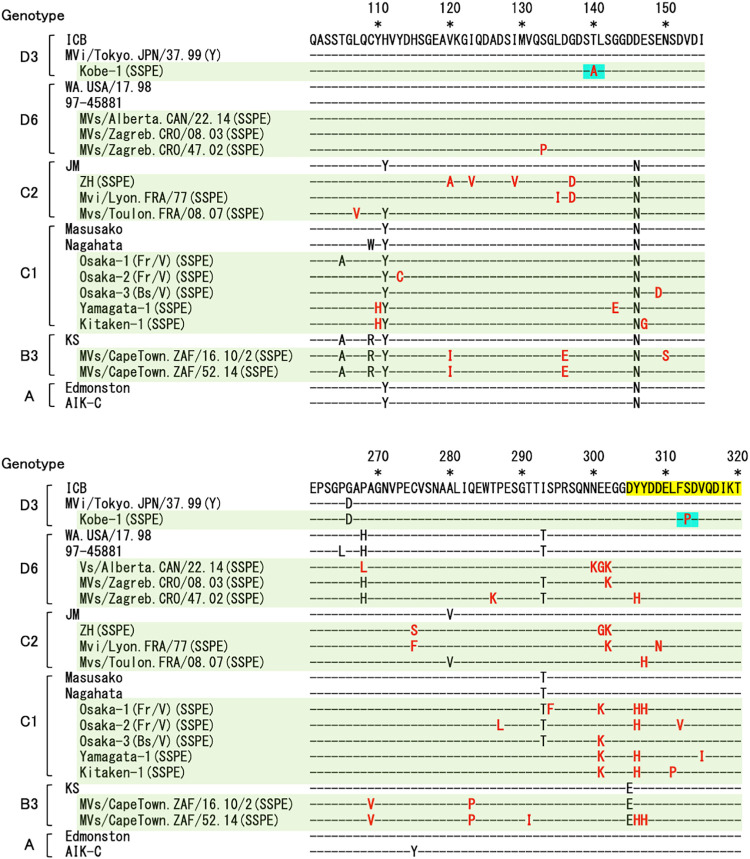
Clusters of amino acid mutations in the P protein of SSPE viruses. Sequence alignment of the region in the P protein surrounding the T140A and S313P mutations (marked in light blue) of the SSPEV Kobe-1 strain. Several strains of MV and SSPE virus of the same genotype (D3, D6, C2, C1, B3 and A) are compared. The red letters indicate mutations specific to SSPE viruses. DM domain of the P protein is marked in yellow. -, equivalent amino acid to that of ICB. Accession numbers in DDBJ/EMBL/GenBank: ICB, AB016162; MVi/Tokyo.JPN/37.99(Y), GQ376026; Kobe-1, AB254456; WA.USA/17.98, DQ227321; 97–45881, DQ227319; MVs/Alberta.CAN/22.14, MF775733; MVs/Zagreb.CRO/08.03, DQ227320; MVs/Zagreb.CRO/47.02, DQ227318; JM, M90469; ZH, AB453187; Mvi/Lyon.FRA/77, HM562908; Mvs/Toulon.FRA/08.07, HM562909; Masusako, LC655230; Nagahata, D63927; Osaka-1/Fr/V, LC655226; Osaka-2/Fr/V, LC655228; Osaka-3/Bs/V, LC655229; Yamagata-1, D10635; Kitaken-1, AB453045; KS, HM439386; MVs/CapeTown.ZAF/16.10/2, KT851526; MVs/CapeTown.ZAF/52.14, KT851534; Edmonston, K01711; AIK-C, AB046218.

The RdRp activity of the SSPEV-P protein progressively decreased as mutations (e.g., T140A and S313P) accumulated. The T140A and S313P mutations are unique to the SSPEV Kobe-1 strain and are not found in the P protein of other sequenced SSPE viruses. Alignment analysis, however, identified clusters of mutations at around amino acid 110–150 and 270–320, in which T140A and S313P are included, respectively (Fig 10). Amino acid 313 is located at the N-terminal end of DM domain of the P protein and mutations accumulated especially around this region. It is of interest whether these mutations in the other SSPE viruses than the Kobe-1 strain alter RdRp activity and are involved in the establishment of the persistent MV infection. We did not find any clusters of mutations in the L protein sequence of SSPE viruses. Mutations in the L protein, S519N, I539V and K601R, were specific to the SSPEV Kobe-1 strain. Comparison of the P and L proteins between the MV vaccine strains (genotype A) and the wild-type viruses revealed no marked characteristics in regards to amino acids with which mutation occurred in SSPE viruses.

It is currently impossible to determine the order in which mutations accumulated in proteins of an SSPE virus. In the L protein of the SSPEV Kobe-1 strain, the K601R, S519N, and I538V mutations were introduced during the very early stage of persistent infection because each mutation decreased RdRp activity to a level below the activity of the SSPEV-L protein. The L(triple) protein had extremely low RdRp activity and enabled the establishment and maintenance of persistent infection; virus possessing the L(triple) protein released few infectious viral particles and exhibited restricted growth in neuronal cells (Fig 6). These changes are followed by reinforcement at the late stage (after the F protein has become hyperfusogenic), which facilitates the spread of infection by cell-to-cell fusion (Fig 9). The reinforcement of RdRp activity was experimentally confirmed by addition of other mutations in the SSPEV-L protein (Fig 7), which corresponded to the enhancement of viral propagation in neuronal cells (Fig 8). In a previous study, we isolated a variant of the SSPE virus Kobe-1 strain that replaced the Kobe-1 strain after long-term passage in human neuronal cells because of accelerated cell-to-cell fusion [56]. The enhanced RdRp activity of the variant increased the expression of viral proteins and conferred robust cell-to-cell fusion ability. Variants of SSPE virus with improved growth in the brain may be selected during disease progression, and the isolation of the Kobe-1 variant suggested that RdRp activation could provide selective pressure. Therefore, MV establishes persistent infection by suppressing its RdRp activity, spreads via cell-to-cell fusion after hyperfusogenic mutation of the F protein, and promotes further viral propagation by enhancing RdRp activity. Fig 9 shows a hypothetical model of the transformation of MV into the SSPE Kobe-1 strain.

The isolation of virus during the transformation of MV into SSPE virus is impractical before the appearance of clinical signs. It is also difficult to repeatedly isolate a series of viruses from a single patient because virus can be isolated only via biopsy or during autopsy [33,44,65–68]. Therefore, we cannot currently trace the accumulation of mutations in the MV genome during disease progression. Although mutations in the SSPE virus genome contain information regarding its transformation from the parental MV, the large number of such mutations may preclude analysis of their effects. Because the Kobe-1 strain was isolated 6 weeks after disease onset from a patient who had contracted acute measles 5 years prior, and thus the virus bears only 49 amino acid substitutions [65], we could chase the effect of each mutation. The mutation process of the L protein we have proposed here is hypothetical, and analyses of other SSPE viruses will provide additional insights.

## Materials and methods

### Ethics statement

Animal experiments were reviewed and approved by the Committee of the Institute for Experimental Animals, Kobe University Graduate School of Medicine (permit number 23–67), and all procedures were performed in accordance with relevant guidelines.

### Cells and viruses

Vero cells constitutively expressing human SLAM (Vero/hSLAM; a gift from Y. Yanagi) [25] were maintained in Roswell Park Memorial Institute (RPMI) 1640 medium supplemented with 8% fetal bovine serum. SH-SY5Y human neuroblastoma cells were maintained in Dulbecco’s modified Eagle’s medium/Ham’s F-12 medium (Wako Pure Chemical, Osaka, Japan) supplemented with 8% fetal bovine serum. Baby hamster kidney fibroblast (BHK) cells constitutively expressing T7 RNA polymerase (BHK/T7-9; a gift from N. Ito and M. Sugiyama) [69] were maintained in high-glucose Dulbecco’s modified Eagle’s medium supplemented with 5% fetal bovine serum, 10% tryptose phosphate broth solution (Sigma-Aldrich, St. Louis, MO, USA), and 0.6 mg/mL hygromycin B. B95a cells (marmoset B cells transformed with Epstein–Barr virus) [70] were maintained in high-glucose Dulbecco’s modified Eagle’s medium supplemented with 10% fetal bovine serum.

The isolation of the SSPE virus Kobe-1 strain was described previously (DDBJ/EMBL/GenBank: AB254456.1) [65]. The Kobe-1 strain was derived from the MV of the genotype D3, and the ICB strain [71] (referred to as the 84–01 strain before [70]) is one of the representative strains of the D3 MV [72]. Recombinant MVs (rMVs) were generated in accordance with the method of Seki *et al*. [73] by transfecting BHK/T7-9 cells with plasmids containing the full-length MV genome described below, as well as three support plasmids: pCITE-IC-N, pCITE-IC-PΔC, and pCITEko-9301B-L (gifts from M. Takeda) [74]. The generated rMVs were propagated in Vero/hSLAM cells, and cell-free infectious rMV particles were collected as described previously for viruses carrying the M gene of SSPE virus. Briefly, the culture medium was replaced with medium containing 1 μM cytochalasin D (Sigma-Aldrich); the cells were incubated overnight at 37°C, and infected cells were scraped and pipetted vigorously. The resulting suspension was stored at –80°C until downstream processing.

T7 RNA polymerase-expressing vaccinia virus (vTF7-3) was a gift from B. Moss [75].

### Plasmid construction

The plasmid p(+)MV323, which contained all MV ICB strain genes, was a gift from K. Takeuchi [71]. The cDNA of the genome of the SSPE virus Kobe-1 strain (GenBank: AB254456) was synthesized by reverse transcription polymerase chain reaction (PCR). The *Sal*I–*Spe*I fragment [nucleotides (nt) 3365–9175 according to the ICB strain genome sequence (GenBank: AB016162)] [76] of p(+)MV323 was replaced with the corresponding region of the Kobe-1 strain, yielding a plasmid with the full-length genome of the ICB strain carrying the M, F, and H genes of the SSPE virus Kobe-1 strain [p(+)MV323/sMFH]. The *Spe*I–*Eco47*III fragment (nt 9176–15767) of p(+)MV323/sMFH was replaced with the corresponding regions of the Kobe-1 strain, yielding a plasmid with the genome of the ICB strain carrying the M, F, H, and L genes of the SSPE virus Kobe-1 strain [p(+)MV323/sMFHL]. The plasmid p(+)MV323/SSPEV containing all genes of the Kobe-1 strain was constructed by replacing the *Sma*I–*Sal*I fragment (nt 839–3364) of p(+)MV323/sMFHL with the corresponding region of the Kobe-1 strain.

The plasmid p(+)MV323-EGFP, which contained all MV ICB strain genes and an additional EGFP transcriptional unit, was a gift from Y. Yanagi [77]. The plasmids p(+)MV323-EGFP/SSPEV, p(+)MV323-EGFP/sM, and p(+)MV323-EGFP/sMFH, which contained all genes, only the M gene, and only the M, F, and H genes of the SSPE virus Kobe-1 strain, respectively, were described previously [54]. To prepare chimeric N–P genes units, the MV-N (nt 98–1826) and MV-P (nt 1807–3369) gene fragments, or the SSPEV-N (nt 98–1826) and SSPEV-P (nt 1807–3369) gene fragments, were amplified by PCR using p(+)MV323-EGFP or p(+)MV323-EGFP/SSPEV as templates. Next, the MV-N and SSPEV-P gene fragments, or the SSPEV-N and MV-P gene fragments, were connected and amplified by overlap extension PCR to obtain chimeric N–P genes units (nt 98–3369). The full-length genome plasmids for generation of various chimeric viruses were constructed by using the BspT104I–*Sal*I fragment (nt 100–3364) or the *Spe*I–*Eco47*III fragment (nt 9176–15767) to replace the N–P genes unit or L gene unit of the MV ICB strain and the SSPE virus Kobe-1 strain, respectively, on the p(+)MV323-EGFP, p(+)MV323-EGFP/SSPEV, p(+)MV323-EGFP/sM, and p(+)MV323-EGFP/sMFH plasmids. To introduce the triple mutations (S519N, I539V, and K601R) or triple plus additional mutations into the L protein of the rMVs, the *Spe*I–*Eco47*III fragment (nt 9176–15767) of the plasmids p(+)MV323-EGFP, p(+)MV323-EGFP/sM, or p(+)MV323-EGFP/sMFH was replaced with the corresponding fragment from pGEM/MV-L(triple) or pGEM/MV-L(triple) with the additional mutations described below.

To construct pCA7/MV-N for expression of the N protein of MV, the N gene fragment (nt 108–1685) was amplified by PCR using p(+)MV323-EGFP and cloned into the pCA7 vector. pCA7/MV-P-ΔCV was constructed by cloning the P gene fragment (nt 1807–3330) of the MV ICB strain with nucleotide substitutions causing C and V protein deletion into the pCA7 vector. The *Spe*I–*Eco47*III fragment of p(+)MV323-EGFP was cloned into the pBluescript II SK (-) vector to obtain pBS/MV-L. Next, the *Eco*RI–*Eag*I fragment of pBS/MV-L was cloned into the pGEM vector to produce pGEM/MV-L for MV-L protein. pCA7/SSPEV-N, pCA7/SSPEV-P-ΔCV, and pGEM/SSPEV-L expressing the N, P, and L proteins of the SSPE virus Kobe-1 strain, respectively, were described previously [56]. To introduce mutations into the P and L proteins of MV, fragments with nucleotide substitutions causing the corresponding amino acid mutations were amplified by PCR using pCA7/MV-P-ΔCV and pGEM/MV-L as templates. Plasmids harboring the mutations were generated using In-Fusion Snap Assembly Master Mix (Takara Bio, Shiga, Japan) based on pCA7/MV-P-ΔCV and pGEM/MV-L, in accordance with the manufacturer’s protocol. pGEM/MV-L(triple), prepared for expression of the L protein with S519N, I539V, and K601R mutations, was used as the template to introduce further mutations in the same manner. pcDNA3/R-Luc was constructed by cloning the *Renilla* luciferase gene fragment into the pcDNA3 vector. Primer sequences used to construct these plasmids are available upon request.

### Virus titration

Monolayers of Vero/hSLAM cells in 24-well plates were infected with serially diluted virus samples. After incubation for 1 h at 37°C, the virus samples were removed, and medium containing 100 μg/mL fusion-inhibiting peptide (4092; Peptide Institute, Osaka, Japan) was added to block secondary infections. After 60 h, spots expressing EGFP were counted using a fluorescence microscope (Axioskop2; Zeiss, Oberkochen, Germany); the number of the fluorescent spots was regarded as plaque-forming unit (PFU) [78].

### Virus challenge

BALB/c suckling mice, purchased from CLEA Japan, Inc. (Tokyo, Japan), were observed for health condition for a week and used prior to the age of 3 weeks. Mice were anesthetized, then intracerebrally inoculated with 7 × 10^2^ PFU of each recombinant chimeric virus in a 20 μL suspension of B95a cells. After inoculation, clinical signs were observed daily, and moribund mice were euthanized.

### Cell-to-cell fusion assay

Vero/hSLAM cells cultured in T25 flasks were infected with cell-free rMVs and incubated at 37°C. At the indicated time points, photographs of representative syncytia were obtained using a fluorescence microscope. The percentage area occupied by syncytia in the images was quantified using ImageJ software (http://imagej.nih.gov/ij/) and expressed as cell-to-cell fusion activity.

### Quantitative PCR (qPCR)

Monolayers of Vero/hSLAM cells in 24-well plates were infected with rMVs at a multiplicity of infection (MOI) of 0.04. After 2 h of incubation at 37°C, the virus samples were removed, and medium containing 100 μg/mL of fusion-inhibiting peptide was added. At 48 h post-infection, total RNA was extracted from virus-infected Vero/hSLAM cells using a NucleoSpin RNA kit (Macherey-Nagel); cDNA was prepared by reverse transcription using ReverTra Ace (Toyobo) in accordance with the manufacturer’s protocol, using specific primers for mRNA, (-) genomic RNA, and (+) genomic RNA. qPCR was performed using FastStart Essential DNA Green Master Mix and LightCycler 96 (Roche Diagnostics, Basel, Switzerland) at 95°C for 600 s, followed by 45 cycles of 95°C for 10 s, 55°C for 20 s, and 72°C for 20 s. Melting curve analysis was performed after amplification. Relative expression levels of viral RNAs were calculated by the ΔΔCt method, using β-actin mRNA as the internal control. Primer sets for the target genes were described previously [56].

### Cell-surface biotinylation and Western blot analysis

Vero/hSLAM cells were infected with rMVs as described above for qPCR. At 48 h post-infection, cells were incubated with 0.5 mg EZ-Link Sulfo-NHS-SS-Biotin (Thermo Fisher Scientific, Waltham, MA, USA) at room temperature for 30 min, then lysed in 1 mL of lysis buffer (5 mM sodium chloride, 0.5% Triton X-100, 0.5% sodium deoxycholate, and 10 mM Tris-hydrochloric acid, pH 7.5) at 4°C for 1 h. Next, lysates were centrifuged at 13,000 × *g* for 10 min at 4°C, and supernatants were collected. A small amount (24 μL) of each cell extract was mixed with sodium dodecyl sulfate (SDS) loading buffer. To detect the M protein, cell extracts were concentrated by chloroform/methanol precipitation using the method of Saito *et al*. [79]. Briefly, 200 μL of cell extract were mixed with 200 μL of methanol and 50 μL of chloroform, then centrifuged at 13,000 × *g* for 5 min at 4°C. The protein pellet was washed with methanol, then mixed with SDS loading buffer. For collection of cell-surface samples, 800 μL of each cell extract were mixed with Streptavidin Sepharose High Performance (GE Healthcare, Chicago, IL, USA) and incubated at 4°C for 2 h. The adsorbed biotinylated protein was used as the cell-surface sample.

Samples were separated by SDS-polyacrylamide gel electrophoresis in 12% polyacrylamide gels, then electroblotted onto polyvinylidene difluoride membranes. Proteins were detected by incubating the membranes with a rabbit polyclonal antibody against MV-F protein [80], mouse monoclonal antibody against MV-M protein (MAB8910; Merck Millipore, Burlington, MA, USA), or mouse monoclonal antibody against β-actin (#3700; Cell Signaling Technology, Danvers, MA, USA). Membranes were then incubated with a horseradish peroxidase-conjugated goat anti-rabbit IgG (#7074; Cell Signaling Technology) or goat anti-mouse IgG (sc-2005; Santa Cruz Biotechnology, Dallas, TX, USA) secondary antibody. Proteins were visualized using ImmunoStar Zeta (Fujifilm Wako Pure Chemical Corp., Tokyo, Japan) and C-DiGit Chemiluminescent Western Blot Scanner (LI-COR, Lincoln, NE, USA).

### Mini-genome assay

Subconfluent monolayers of Vero/hSLAM cells in 24-well plates were transfected with 80 ng of the N protein-expressing pCA7/MV-N or pCA7/SSPEV-N; 120 ng of the P protein-expressing pCA7/MV-P-ΔCV, pCA7/SSPEV-P-ΔCV, or pCA7/MV-P-ΔCV with a mutation; 300 ng of the L protein-expressing pGEM/MV-L, pGEM/SSPEV-L, or pGEM/MV-L with mutations; and 350 ng of the MV mini-genome plasmid p18MGFLuc01 encoding the firefly luciferase gene (a gift from K. Komase) [81], along with 10 pg of the *Renilla* luciferase-expressing pcDNA3/R-Luc, then incubated at 37°C. At 24 h post-transfection, luciferase activity was measured using the Dual-Luciferase Reporter Assay System (Promega, Madison, WI, USA) and Centro XS3 LB960 (Berthold Technologies, Bad Wildbad, Germany) in accordance with the manufacturers’ protocols. Relative RdRp activity was calculated through division of firefly luciferase activity by *Renilla* luciferase activity. In Fig 4A, the value in the absence of N, P, and L proteins was shown as the control. In other Figs, the results were demonstrated with the value after subtracting the value of control (in the absence of N, P, and L proteins).

### Infectious virus particle assay

Monolayers of Vero/hSLAM cells in 24-well plates were infected with cell-free rMVs at a MOI of 0.04. After 2 h of incubation at 37°C, virus samples were removed; the cells were washed with phosphate-buffered saline and incubated at 37°C. The culture medium was collected and replaced with fresh medium every 24 h. After the medium had been centrifuged at 2,000 × *g* for 5 min at 4°C, the numbers of infectious cell-free viruses in supernatant were measured as described above for virus titration.

### Statistical analysis

Comparisons between two groups were conducted using unpaired two-tailed Student’s t-tests. P-values < 0.05 were considered indicative of statistical significance.

We performed the log-rank tests using the survival package in the R Software to analyze differences between survival curves. If a p-value was less than 0.05, the difference between the survival curves was considered statistically significant.

## Supporting information

S1 Fig The SSPEV-L gene and the SSPEV-N and/or SSPEV-P genes suppressed cell-to-cell fusion and propagation of rMVs in neuronal cells. (**A**) Enlargement of syncytia formed by rMV-infected Vero/hSLAM cells. Vero/hSLAM cells were infected with the EGFP-expressing cell-free rMVs in Fig 2A and a syncytium derived from a single infected cell was observed at 12-h intervals under a fluorescence microscope. A representative photograph at each time point is presented. Magnification, ×200. mock, uninfected cells. n.a., not applicable. (**B**) Spread of rMV infection in human neuronal cells. SH-SY5Y cells were infected with the EGFP-expressing cell-free rMVs in Fig 2A and the spread of infection from a single infected cell was observed at 12-h intervals under a fluorescence microscope. A representative photograph at each time point is presented. Magnification, ×200. mock, uninfected cells. n.a., not applicable.
(TIFF)

S2 Fig Suppression of RdRp activity is associated with the reduction of viral cell-to-cell fusion and growth in neuronal cells. (**A**) Schematic of the genomes of the EGFP-expressing rMV/sMFH variants after exchanges of N, P, and L genes between the MV ICB strain and the SSPE virus Kobe-1 strain. (**B**) Viral cell-to-cell fusion. Vero/hSLAM cells were infected with the rMVs in (A). After incubation at 37°C for 36 h, a syncytium derived from a single infected cell was observed and photographed under a fluorescence microscope. Magnification, ×200. Cell-to-cell fusion was quantified as in Fig 2B. Data from five images are shown as means ± standard deviations. (**C**) Viral propagation in human neuronal cells. SH-SY5Y cells were infected with the rMVs in (A). After incubation at 37°C for 72 h, the spread of infection from a single infected cell was observed and photographed under a fluorescence microscope. Magnification, ×200. Spread of viral infection was determined as in Fig 2C. Data from five images are shown as means ± standard deviations.
(TIFF)

S3 Fig The L(triple) protein restricted the release of infectious virus particles. Vero/hSLAM cells were infected with rMVs bearing the MV-M protein (**A**) or the SSPEV-M protein (**B**) in Fig 6A, then incubated at 37°C. Culture medium was collected every 24 h until 4 days post-infection. Cell-free viruses in the supernatant after centrifugation were titrated in Vero/hSLAM cells. Data from three independent experiments are shown as means ± standard deviations.
(TIFF)

S4 Fig Restoration of RdRp activity improved viral RNA production and expression of F protein on the cell surface. (**A**) Viral RNA levels in cells infected with rMVs harboring the L(triple) gene and its variants. Vero/hSLAM cells were infected with the rMVs in Fig 8A, and viral mRNA and genomic RNA were quantified as in Fig 3A. Data from three independent experiments are shown as means ± standard deviations. (-), negative-sense genome; (+), positive-sense genome. (**B**) Surface F protein expression of cells infected with rMVs. Vero/hSLAM cells were infected with the rMVs in Fig 8A, cell-surface proteins were biotinylated to detect F protein, and viral proteins were quantified as in Fig 3B. A representative image of several experiments is shown (left panel). Inactive F0 and active F1 forms of the F protein are shown; molecular markers are indicated on right. CS, cell-surface fraction; WC, whole-cell fraction. Intensities of F1, N, and M protein bands were quantified using ImageJ (right panel). Fs, F1 protein in CS; F, F1 protein in WC.
(TIFF)

S5 Fig Cell-to-cell fusion and viral propagation in neuronal cells were improved by restoration of RdRp activity. (**A**) Representative photographs of viral cell-to-cell fusion. Vero/hSLAM cells were infected with the rMVs in Fig 8A and enlargement of fused cells was monitored under a fluorescence microscope; photographs were obtained at 12-h intervals. Magnification, ×200. n.a., not applicable. (**B**) Representative photographs of viral propagation in neuronal cells. SH-SY5Y cells were infected with the rMVs in Fig 8A and the spread of infection from a single infected cell was observed under a fluorescence microscope; photographs were obtained at 12-h intervals. Magnification, ×200. n.a., not applicable.
(TIFF)

S1 Table Amino acid substitutions of the L(triple) protein and its variants in Fig 7. (XLSX)
