# Supplementary figures and images for: Suppression of viral RNA polymerase activity is necessary for persistent infection during the transformation of measles virus into SSPE virus
Source: PLoS Pathog. 2023 Jul 26;19(7):e1011528. doi: 10.1371/journal.ppat.1011528 (PMC10406308; doi:10.1371/journal.ppat.1011528)

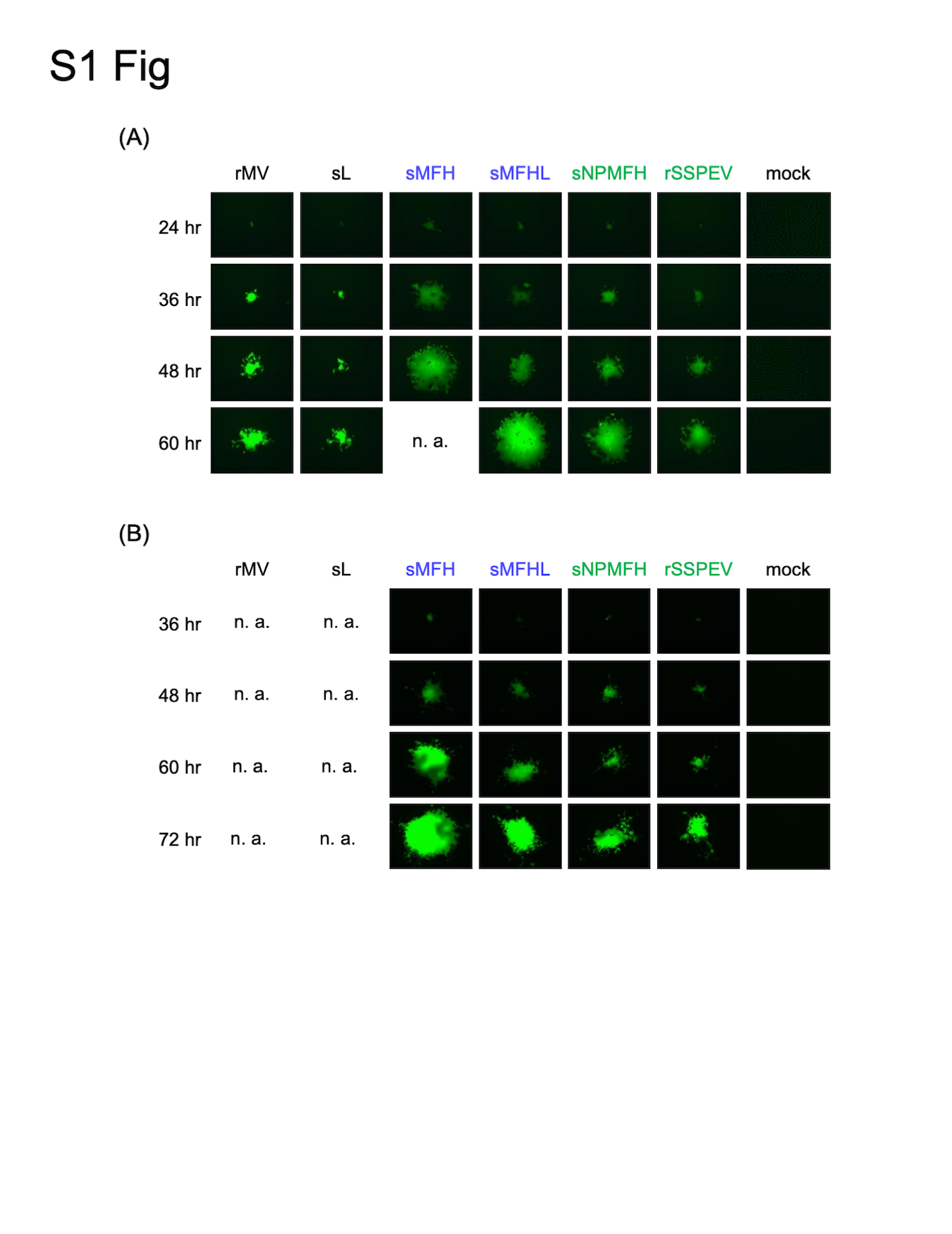

Supplement: S1 Fig — (A) Enlargement of syncytia formed by rMV-infected Vero/hSLAM cells. Vero/hSLAM cells were infected with the EGFP-expressing cell-free rMVs in Fig 2A and a syncytium derived from a single infected cell was observed at 12-h intervals under a fluorescence microscope. A representative photograph at each time point is presented. Magnification, ×200. mock, uninfected cells. n.a., not applicable. (B) Spread of rMV infection in human neuronal cells. SH-SY5Y cells were infected with the EGFP-expressing cell-free rMVs in Fig 2A and the spread of infection from a single infected cell was observed at 12-h intervals under a fluorescence microscope. A representative photograph at each time point is presented. Magnification, ×200. mock, uninfected cells. n.a., not applicable. (TIFF) [file ppat.1011528.s001.tiff]

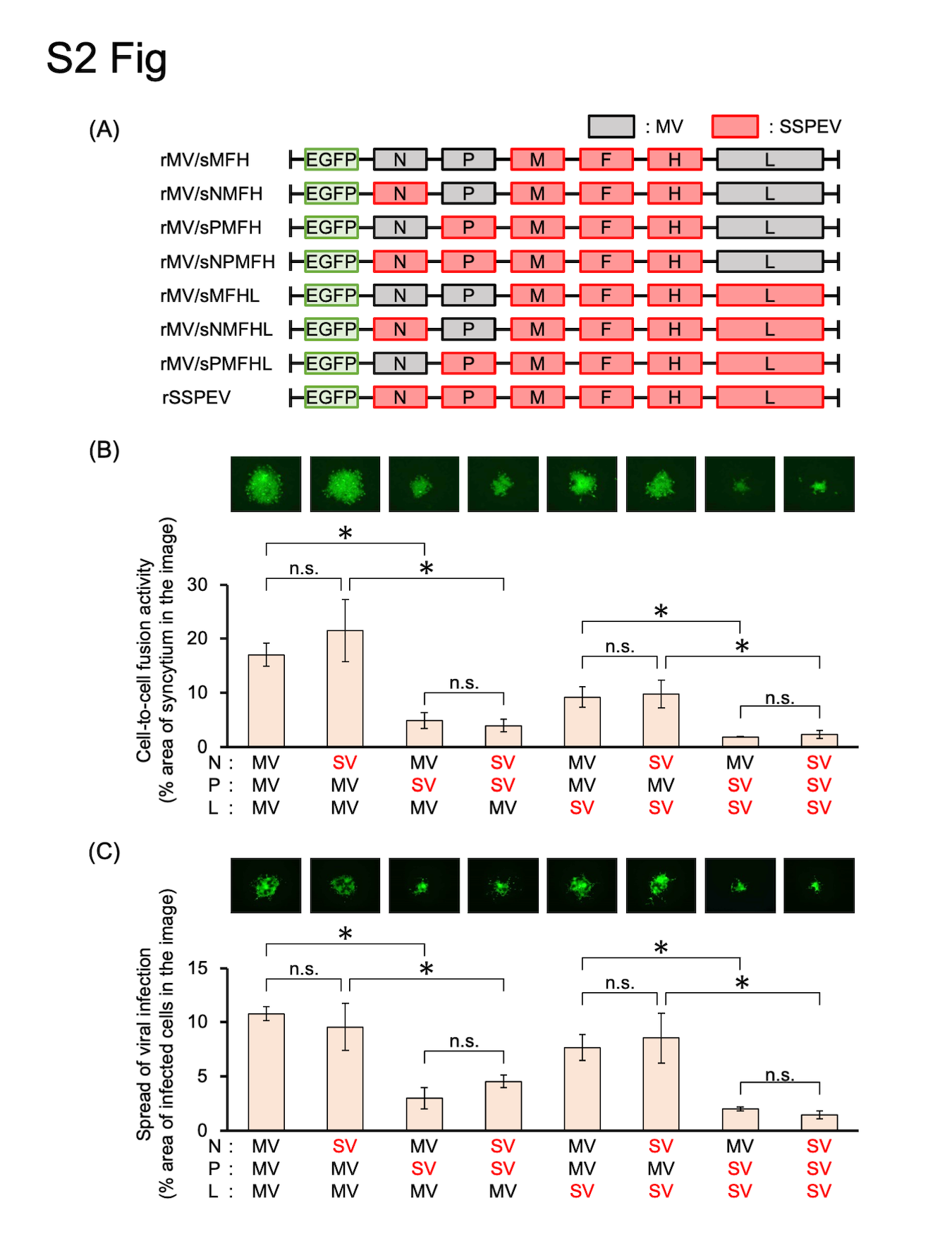

Supplement: S2 Fig — (A) Schematic of the genomes of the EGFP-expressing rMV/sMFH variants after exchanges of N, P, and L genes between the MV ICB strain and the SSPE virus Kobe-1 strain. (B) Viral cell-to-cell fusion. Vero/hSLAM cells were infected with the rMVs in (A). After incubation at 37°C for 36 h, a syncytium derived from a single infected cell was observed and photographed under a fluorescence microscope. Magnification, ×200. Cell-to-cell fusion was quantified as in Fig 2B. Data from five images are shown as means ± standard deviations. (C) Viral propagation in human neuronal cells. SH-SY5Y cells were infected with the rMVs in (A). After incubation at 37°C for 72 h, the spread of infection from a single infected cell was observed and photographed under a fluorescence microscope. Magnification, ×200. Spread of viral infection was determined as in Fig 2C. Data from five images are shown as means ± standard deviations. (TIFF) [file ppat.1011528.s002.tiff]

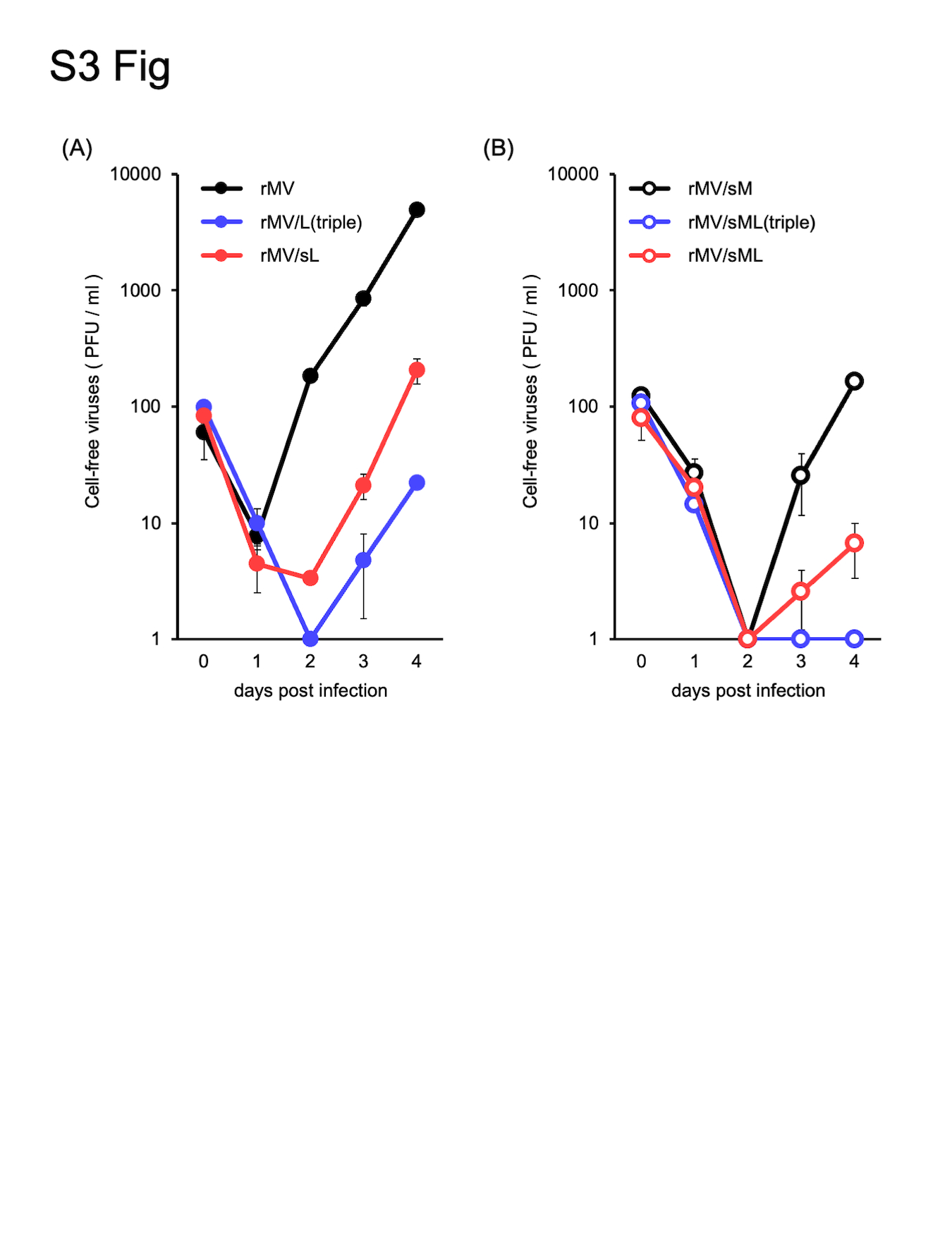

Supplement: S3 Fig — Vero/hSLAM cells were infected with rMVs bearing the MV-M protein (A) or the SSPEV-M protein (B) in Fig 6A, then incubated at 37°C. Culture medium was collected every 24 h until 4 days post-infection. Cell-free viruses in the supernatant after centrifugation were titrated in Vero/hSLAM cells. Data from three independent experiments are shown as means ± standard deviations. (TIFF) [file ppat.1011528.s003.tiff]

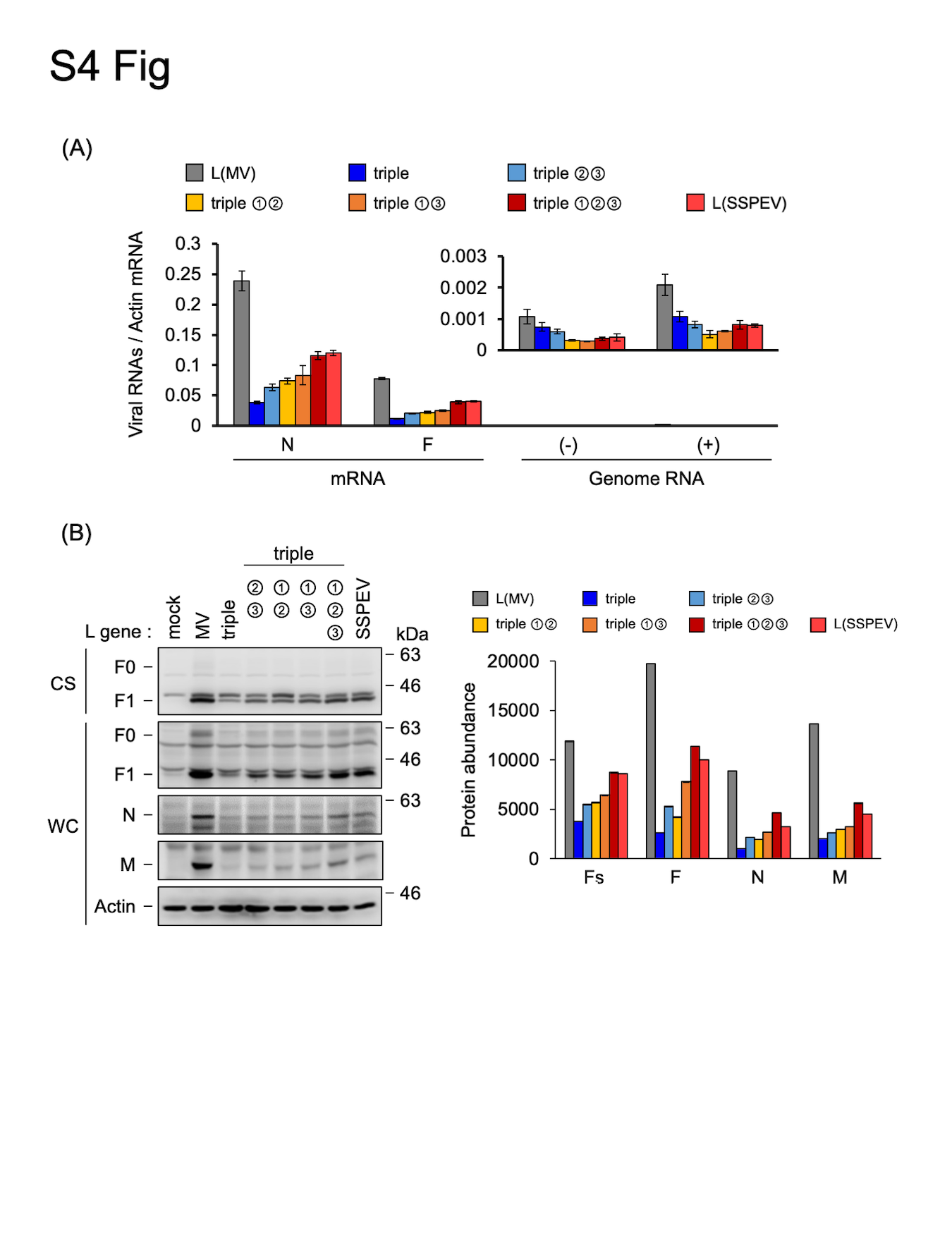

Supplement: S4 Fig — (A) Viral RNA levels in cells infected with rMVs harboring the L(triple) gene and its variants. Vero/hSLAM cells were infected with the rMVs in Fig 8A, and viral mRNA and genomic RNA were quantified as in Fig 3A. Data from three independent experiments are shown as means ± standard deviations. (-), negative-sense genome; (+), positive-sense genome. (B) Surface F protein expression of cells infected with rMVs. Vero/hSLAM cells were infected with the rMVs in Fig 8A, cell-surface proteins were biotinylated to detect F protein, and viral proteins were quantified as in Fig 3B. A representative image of several experiments is shown (left panel). Inactive F0 and active F1 forms of the F protein are shown; molecular markers are indicated on right. CS, cell-surface fraction; WC, whole-cell fraction. Intensities of F1, N, and M protein bands were quantified using ImageJ (right panel). Fs, F1 protein in CS; F, F1 protein in WC. (TIFF) [file ppat.1011528.s004.tiff]

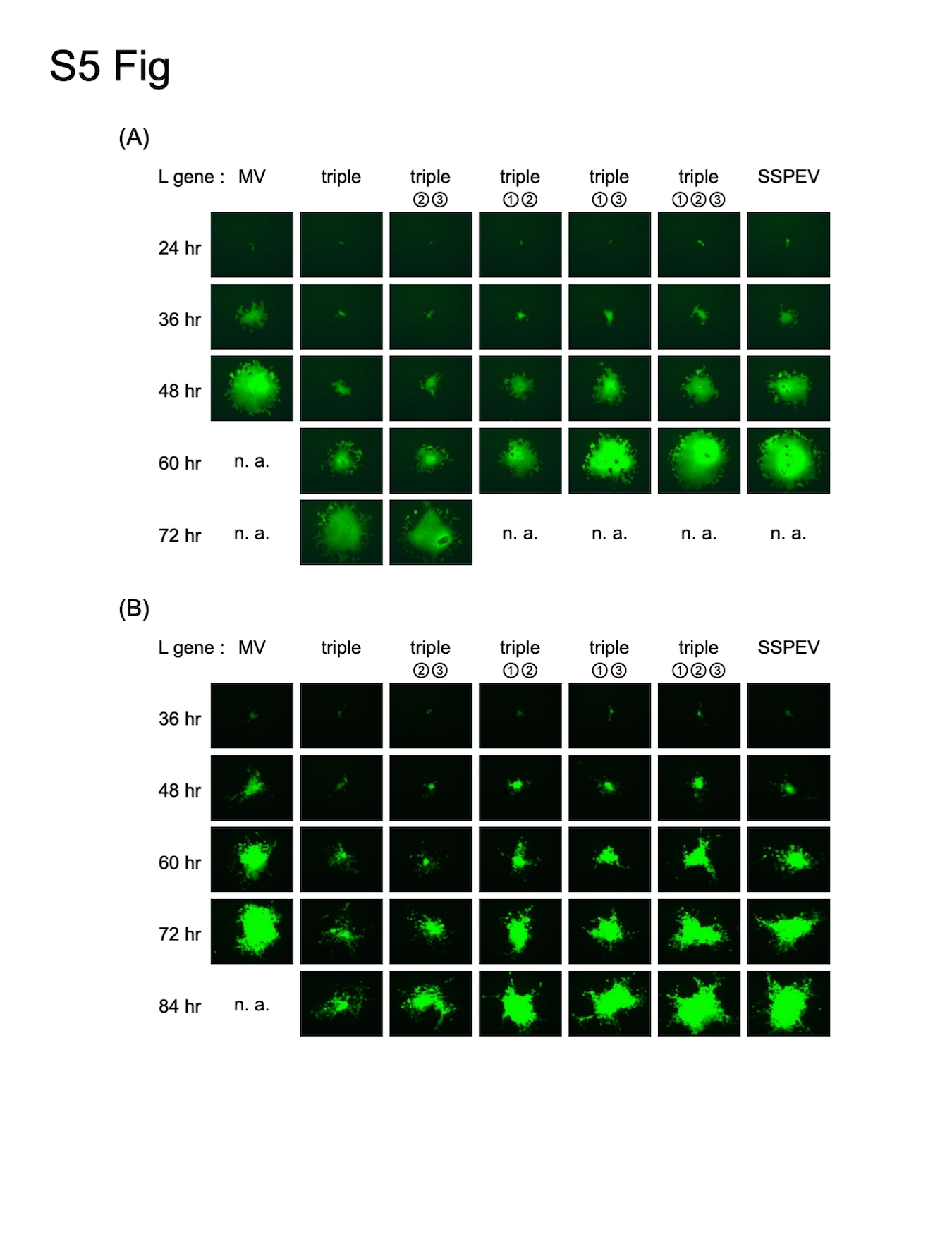

Supplement: S5 Fig — (A) Representative photographs of viral cell-to-cell fusion. Vero/hSLAM cells were infected with the rMVs in Fig 8A and enlargement of fused cells was monitored under a fluorescence microscope; photographs were obtained at 12-h intervals. Magnification, ×200. n.a., not applicable. (B) Representative photographs of viral propagation in neuronal cells. SH-SY5Y cells were infected with the rMVs in Fig 8A and the spread of infection from a single infected cell was observed under a fluorescence microscope; photographs were obtained at 12-h intervals. Magnification, ×200. n.a., not applicable. (TIFF) [file ppat.1011528.s005.tiff]
